# Investigating old‐growth ponderosa pine physiology using tree‐rings, δ^13^C, δ^18^O, and a process‐based model

**DOI:** 10.1002/ecy.2656

**Published:** 2019-04-15

**Authors:** Danielle E. M. Ulrich, Christopher Still, J. Renée Brooks, Youngil Kim, Frederick C. Meinzer

**Affiliations:** ^1^ Bioscience Division Los Alamos National Laboratory P.O. Box 1663 MS M888 Los Alamos New Mexico 87545 USA; ^2^ Department of Forest Ecosystems and Society Oregon State University Corvallis Oregon 97331 USA; ^3^ Western Ecology Division US EPA/NHEERL Corvallis Oregon 97331 USA; ^4^ USDA Forest Service, Pacific Northwest Research Station Corvallis Oregon 97331 USA

**Keywords:** carbon isotope ratios, effective path length, oxygen isotope ratios, Physiological Principles in Predicting Growth, process‐based modeling, tree rings

## Abstract

In dealing with predicted changes in environmental conditions outside those experienced today, forest managers and researchers rely on process‐based models to inform physiological processes and predict future forest growth responses. The carbon and oxygen isotope ratios of tree‐ring cellulose (δ^13^C_cell_, δ^18^O_cell_) reveal long‐term, integrated physiological responses to environmental conditions. We incorporated a submodel of δ^18^O_cell_ into the widely used Physiological Principles in Predicting Growth (3‐PG) model for the first time, to complement a recently added δ^13^C_cell_ submodel. We parameterized the model using previously reported stand characteristics and long‐term trajectories of tree‐ring growth, δ^13^C_cell_, and δ^18^O_cell_ collected from the Metolius AmeriFlux site in central Oregon (upland trees). We then applied the parameterized model to a nearby set of riparian trees to investigate the physiological drivers of differences in observed basal area increment (BAI) and δ^13^C_cell_ trajectories between upland and riparian trees. The model showed that greater available soil water and maximum canopy conductance likely explain the greater observed BAI and lower δ^13^C_cell_ of riparian trees. Unexpectedly, both observed and simulated δ^18^O_cell_ trajectories did not differ between the upland and riparian trees, likely due to similar δ^18^O of source water isotope composition. The δ^18^O_cell_ submodel with a Peclet effect improved model estimates of δ^18^O_cell_ because its calculation utilizes 3‐PG growth and allocation processes. Because simulated stand‐level transpiration (*E*) is used in the δ^18^O submodel, aspects of leaf‐level anatomy such as the effective path length for transport of water from the xylem to the sites of evaporation could be estimated.

## Introduction

Process‐based tree growth models incorporate physiological principles that enable them to be widely applied to diverse species and sites, in contrast to empirical growth and yield models. This improves our understanding of how variable environmental conditions influence forest productivity and stand characteristics (Landsberg 2003). A widely used stand‐level process model is Physiological Principles in Predicting Growth (3‐PG), developed by Landsberg and Waring (1997) and since modified by numerous other investigators (Xenakis et al. 2008, Gonzalez‐Benecke et al. 2014, Wei et al. 2014a, Almeida and Sands 2016, Forrester and Tang 2016, Meyer et al. 2018). The 3‐PG model utilizes environmental conditions, stand characteristics, and species‐specific physiological and allometric measurements to accurately predict growth and productivity in changing environmental conditions and on diverse forested stands (Coops et al. 1998, 2007, Law et al. 2000, Waring and Gao 2016). 3‐PG uses a simple light‐use efficiency relationship to estimate carbon assimilation (i.e., gross primary productivity, GPP) and the original model version assumes a constant fraction of GPP (0.47) is allocated to net primary productivity (NPP), which is then partitioned into below‐ and aboveground biomass. The flexibility and simplicity of 3‐PG make it advantageous and practical to use for diverse forest types and management applications while also allowing mechanistic processes to be easily examined.

Because carbon allocation is downstream of carbon assimilation, Wei et al. (2014a) used the carbon isotope composition (δ^13^C) of tree rings as a new way to constrain 3‐PG at the carbon assimilation step to more accurately represent allocation processes, thus improving 3‐PG estimates of stand characteristics. This improvement occurred because the tree‐ring δ^13^C signal represents a balance between carbon and water fluxes during the assimilation process, enabling these fluxes to be integrated and mutually constrained within the model. The tree‐ring δ^13^C signal is related to both stomatal conductance (*g*_s_) and photosynthesis (*A*), which together determine the ratio of intercellular to ambient [CO_2_], and consequently ^13^C discrimination (Farquhar et al. 1989). Thus, tree‐ring δ^13^C is commonly used to reconstruct gas exchange and physiological responses to environmental conditions (McCarroll and Loader 2004, Gessler et al. 2014).

In conjunction with the δ^13^C of tree‐ring cellulose (δ^13^C_cell_), the δ^18^O of tree‐ring cellulose (δ^18^O_cell_) has also been used to tease apart the relative contributions of *A* and *g*_s_ to the δ^13^C_cell_ signal because the δ^18^O_cell_ signal primarily reflects water fluxes and processes affecting *g*_s_. Relying on a combination of both δ^13^C_cell_ and δ^18^O_cell_ is known as a “dual isotope approach” and is a useful proxy for leaf‐level gas exchange (Scheidegger et al. 2000, Barnard et al. 2012). Together, tree‐ring δ^13^C_cell_ and δ^18^O_cell_ can shed light on physiological responses to environmental conditions such as drought, temperature, relative humidity, fertilization, thinning, and pests (Williams et al. 2010, Brooks and Mitchell 2011, Marias et al. 2014, Saffell et al. 2014, Voelker et al. 2014a, 2014b, Hartl‐Meier et al. 2015). Therefore, the combination of a tree‐ring δ^18^O_cell_ submodel with the existing δ^13^C_cell_ submodel (Wei et al. 2014a) can reveal more information about stand carbon dynamics and water use and improve our understanding of mechanisms underlying physiological responses to environmental variability.

However, our understanding of the mechanistic drivers of tree‐ring δ^18^O_cell_ is incomplete (Gessler et al. 2014, Treydte et al. 2014). Because 3‐PG predicts growth and allocation (e.g., gross primary productivity [GPP], basal area, basal area increment [BAI], diameter at breast height [DBH], height) as modified by soil water balance and climate variations, the incorporation of a δ^18^O_cell_ submodel into 3‐PG can reveal processes that underlie δ^18^O_cell_ predictions, as previous models of δ^18^O_cell_ have not explicitly included variation in growth or allocation in their predictions (Barbour and Farquhar 2000, Roden et al. 2000). The 3‐PG model also provides monthly estimates of transpiration (*E*) required to determine the Peclet effect, which describes the mixing of isotopically unenriched water arriving at the evaporative sites via bulk flow driven by *E* with the isotopically enriched water diffusing back from the evaporative sites (Farquhar and Lloyd 1993). Although the Peclet effect has improved δ^18^O_cell_ predictions in multiple systems (Barbour et al. 2000, 2004, Holloway‐Phillips et al. 2016), the importance of the Peclet effect for estimating δ^18^O has been debated (Ripullone et al. 2008, Xiao et al. 2012, Loucos et al. 2015, Song et al. 2015, Bögelein et al. 2017). This is partly because contributions of each Peclet effect component are challenging to disentangle, especially the effective path length (*L*), an elusive component of the Peclet effect that is not directly measurable (Song et al. 2013, Loucos et al. 2015). 3‐PG with the δ^18^O_cell_ submodel can advance our understanding of factors that drive δ^18^O_cell_ dynamics including estimates of *L*.

The aim of this study is to test a further modification of 3‐PG that includes both a δ^13^C_cell_ and δ^18^O_cell_ submodel to understand physiological drivers of stand characteristics using long‐term trajectories of tree‐ring growth, δ^13^C_cell_, and δ^18^O_cell_ from old‐growth *Pinus ponderosa* at the AmeriFlux Metolius site in the Oregon Cascades. This study builds upon previous application of 3‐PG to *P. ponderosa* (Law et al. 2000, Coops et al. 2005, Wei et al. 2014b) and is well suited to evaluate how the δ^13^C_cell_ and δ^18^O_cell_ submodels in 3‐PG can improve our understanding of physiological drivers of tree‐ring isotope ratios. For this work, we draw upon a combination of tree‐ring growth and stable isotope observations, along with extensive meteorological and physiological measurements recorded at this site (Law et al. 2000, 2001a, Warren et al. 2005). We parameterized the model using an “upland” set of old‐growth *P. ponderosa* trees ~1 km from the Metolius River located at the US‐Me2 AmeriFlux site. We then tested the parameterized model on a nearby “riparian” set of similarly aged old‐growth trees closer (~0.015 km) to the Metolius River to examine potential site and physiological factors driving observed differences in BAI and δ^13^C_cell_ between these two sites. The goals of this study were to (1) use long‐term measured BAI, δ^13^C_cell_, and δ^18^O_cell_ trajectories to evaluate the performance of the updated 3‐PG model with the δ^13^C_cell_ and δ^18^O_cell_ submodels; (2) use this first test of 3‐PG with a δ^18^O_cell_ submodel to improve our understanding of the mechanistic controls of δ^18^O_cell_; and (3) demonstrate how the model can be used to explore potential site and physiological differences between upland and riparian sets of trees.

## Materials and Methods

### Study site

This study was conducted in a *P. ponderosa* forest on the eastern side of the Cascade Mountains in central Oregon within the Metolius Research Natural Area (US‐Me2, 44.4957° N, 121.6224° W) at an elevation of 915 m. The site consists of 27% old trees (~250 yr), 25% younger trees (~45 yr), and 48% mixed‐age trees. Bitterbrush (*Purshia tridentata*) and bracken fern (*Pteridium aquilinum*) comprise the sparse understory. Precipitation is greatest between October and June with dry summer months. Winters are wet and cold, snow cover in winter is common, and freezing temperatures occur mostly at night and early morning. Soil is classified as sandy loam (73% sand, 21% silt, and 6% clay) and soil nutrients are low (Law et al. 2000, Warren et al. 2005).

### Climate data

AmeriFlux CDIAC climate and eddy flux (ecosystem gross primary productivity, GPP; ecosystem water vapor flux, LE) data for the intermediate ponderosa pine site (US‐Me2) were available for 2002–2012. To extrapolate back in time, monthly minimum and maximum temperature (*T*_min_ and *T*_max_, respectively) obtained from PRISM (http://www.prism.oregonstate.edu/) for 1895–2012 was compared with the site‐level AmeriFlux data and that relationship (*y* = 1.27*T*_min_PRISM_ + 2.46, *R* ^2^ = 0.92; *y* = 0.89*T*_max_PRISM_ − 1.42, *R* ^2^ = 0.98) was used to correct PRISM data, which are based on 4‐km grid cells (data *available online*).^1^ Precipitation data were also obtained from PRISM. As PRISM climate data only extended to 1895, we focused on 1895–2002 in this study. Data on atmospheric [CO_2_] and its δ^13^C from Francey et al. (1999) were used in simulations of this period.

Appendix S1: Fig. S1 describes the required driving climate variables for 3‐PG: annual average minimum air temperature (*T*_min_), average air temperature (*T*_av_), maximum air temperature (*T*_max_), vapor pressure deficit (VPD), solar radiation, and precipitation from 1895 to 2002 (actual mean monthly climate data were used in the model, not the annual averages depicted in Appendix S1: Fig. S1). Mean monthly VPD was calculated as the difference between saturation vapor pressure at minimum and maximum temperature. Mean daily solar radiation was calculated from mean monthly *T*_min_ and *T*_max_ (Bristow and Campbell 1984, Thornton et al. 1997, Coops et al. 1998, 2000) as in Landsberg et al. (2003). The number of frost days per month (*F*) was calculated based on mean monthly *T*_min_:

(1) $$F=T_{\min}\times \left(-2\right)+11.6.$$

If *T*_min_ > 6, then *F* was set to zero. This equation assumes photosynthesis does not occur on days with temperatures below −2°C (Waring 2000).

### Tree‐ring analyses

Tree cores were collected in early spring 2003 from two sets of trees: an upland set ~1 km from the Metolius River at the AmeriFlux Metolius Intermediate Pine site (US‐Me2), and a nearby riparian set within 0.015 km of the Metolius River located just north of Camp Sherman. The upland and riparian sites were <5 km apart. We sampled five upland and five riparian *P. ponderosa* trees of approximately the same stem diameter at 1.3 m height (upland, 87.8 ± 3.9 cm; riparian, 112.3 ± 8.2 cm) and age (mean age ≈ 260 yr; Table 1). In spring 2003 prior to diameter growth, three 12 mm cores from each tree were collected for isotopic analysis, along with a separate 5 mm core that was collected as an archive. Cores were dried and sanded, and the 5 mm core was mounted. All cores were age dated, and ring widths were measured using a sliding stage incremental micrometer (Velmex, Bloomfield, New York, USA) with Measure J2X software (VoorTech Consulting, Holderness, New Hampshire, USA). Visual cross‐dating was verified using the COFECHA program to identify false or missing rings (Holmes 1983) for all cores collected. Tree‐ring widths were converted to basal area increment (BAI, cm^2^·tree^−1^·yr^−1^) by assuming a circular outline of stem cross‐sections.

**Table 1:** Measured and previously reported stand characteristics for old‐growth *Pinus ponderosa* at the Metolius AmeriFlux (upland) site used to parameterize the model. Values for δ^13^C and δ^18^O are 1895–2002 means. Values are means ± SE

Measurement	Value	Reference
Tree height (m)	30.9 ± 0.93	Youngblood et al. (2004)
	33.5 ± 1.26	Law et al. (2001b)
	34 ± 0.8	Law et al. (2001a)
Age (yr)	~260	this study
Diameter at breast height (cm)	upland, 87.8 ± 3.9; riparian, 112.3 ± 8.2	this study
δ^13^C_cell_ (‰)	upland, −23.1 ± 0.07; riparian, −23.8 ± 0.04	this study
δ^18^O_cell_ (‰)	upland, 28.3 ± 0.1; riparian, 28.9 ± 0.1	this study
LAI (m^2^/m^2^)	<1.0	Ryan et al. (2000)
	0.89–1.6	Law et al. (2001b)
	1.1–1.8	Law et al. (2000)
	2.1	Irvine et al. (2002)
Basal area (m^2^/ha)	30	Youngblood et al. (2004), Warren et al. (2005)
	35	Zhang et al. (2013)
	45	Meyer (1938)
Stand density (trees/ha)	54	Youngblood et al. (2004)
	72	Law et al. (2001a), Warren et al. (2005)
	84	Law et al. (2001b)
	137	Meyer (1938)
Net primary productivity (t dry mass·ha^−1^·yr^−1^)	9.2	Law et al. (2000)

### Sample preparation

The 12 mm cores were separated into annual increments spanning from 2002 to 1850 (152 yr). However, we focused on 1895–2002 in this study because PRISM climate data only went back to 1895. The annual increments from three cores per tree were combined for a single sample per tree per year. Each annual ring was ground with a ball mill to a fine powder. All samples were extracted for alpha‐cellulose. Oils and resins were removed with toluene‐ethanol and ethanol Soxhlet extractions (Leavitt and Danzer 1993). Holocellulose was isolated by delignification in an acetic acid‐acidified sodium chlorite solution and converted to alpha‐cellulose in sodium hydroxide (Sternberg 1989).

Approximately 0.8 mg of alpha‐cellulose was loaded into tin capsules for C combustion and 0.4 mg into silver capsules for O pyrolysis for subsequent isotopic analysis by isotope ratio mass spectrometer (IRMS) at the Integrated Stable Isotope Research Facility at the Western Ecology Division of the U.S. EPA, Corvallis Oregon, USA. Samples analyzed for ^13^C were flash combusted using an elemental analyzer (ECS 4010; Costech, Valencia, California, USA), and the resulting CO_2_ analyzed by continuous‐flow IRMS (Delta Plus XP, Finnigan MAT, now Fisher Scientific, Waltham, MA, USA). Each run was calibrated using three internal standards (NIST concentration standards of corn, bovine liver, and tomato) spanning the range of expected values, with an independent QC standard (cellulose) to calculate accuracy. Internal standards were routinely calibrated to international standards USGS42 (Tibetan hair), NIST 8542 sucrose, NIST 8573 and 8574 glutamic acid, and NIST 8514 graphite. Typical precision and accuracy was ±0.1‰ or better as determined by repeated measures of internal quality control standards and from sample replicates. Samples were analyzed for ^18^O using a high temperature conversion elemental analyzer (TC/EA ThermoQuest Finnigan, now Fisher Scientific) interfaced to an IRMS (Thermo Electron Delta XL, now Fisher Scientific). Internal laboratory standards (NIST concentration standards of pine needles, sucrose, and corn) were used for calibration standards with an independent QC standard (cellulose) for accuracy estimates. IAEA‐601 and IAEA‐602 benzoic acid were used to routinely calibrate the internal standards. Typical error was ±0.2‰ or better as determined by repeated measures of internal quality control standards and from sample replicates. The C and O stable isotope ratios (*R*) of the heavier (i.e., ^13^C, ^18^O) to lighter isotope (i.e., ^12^C, ^16^O) were represented by delta (δ) notation in parts per thousand (‰) relative to the VPDB or VSMOW international standards (McCarroll and Loader 2004):

(2) $$\delta^{13}C\;\text{or}\;\delta^{18}O=\left(\frac{R_{\mathrm{sample}}}{R_{\mathrm{standard}}}-1\right)‰.$$

### 3‐PG isotope model

3‐PG is a simplified model that incorporates essential tree physiological, hydrologic, and growth processes to predict net primary productivity (NPP), biomass allocation, water use, soil water balance, stem mortality (self‐thinning), litterfall, and root turnover on a monthly time step (Landsberg and Waring 1997). The input/driving data include mean monthly values of *T*_min_, *T*_max_, *T*_av_, precipitation, *F*, solar radiation, VPD, atmospheric [CO_2_], δ^13^C of the atmosphere, and δ^18^O of source water. Stand initialization values include initial foliage biomass, root biomass, stem biomass, stand density (stocking), available soil water, and maximum available soil water and were based on previously reported values (Appendix S1: Table S1). The stand‐level outputs include biomass pools for roots, stems, and foliage, GPP, NPP, transpiration (*E*), growth and stand characteristics, and δ^13^C_cell_ and δ^18^O_cell_ in this study. In all figures and tables, outputs are presented as averages for May–September when *P. ponderosa* physiological activity and radial growth at this site occurs (Fowells 1941).

3‐PG is based on the light‐use efficiency modeling paradigm that captures a positive linear relationship between plant growth and intercepted radiation. Specifically, 3‐PG calculates GPP from absorbed photosynthetically active radiation (ϕ_p.a_, mol/m^2^) and canopy quantum efficiency (α_c_, mol C/mol photon) and is constrained by factors that influence stomatal closure, including atmospheric VPD, soil moisture, temperature, frost, and site nutrient status:

(3) $$\text{GPP}=\alpha_{c}\phi_{p.a}\approx \alpha_{\mathrm{cx}}\phi_{p.a}f_{T}f_{F}f_{N}f_{D}f_{\Theta }f_{\mathrm{age}}$$

where *f*_T_, *f*_F_, *f*_N_, *f*_D_, *f*_ϴ_, and *f*_age_ are the temperature, frost, nutrition, VPD, soil water, and age modifiers, respectively, and α_cx_ is the maximum canopy quantum efficiency.

The temperature modifier (*f*_T_) incorporates the minimum, maximum, and optimum temperatures for growth. The frost modifier (*f*_F_) is calculated using *F*. The nutrient modifier (*f*_N_) is a function of site fertility rating (FR), ranging from 0 to 1, and is based on available soil nutrients. The VPD modifier (*f*_D_) is a function of *k*_g_, a species‐specific coefficient describing the strength of the response of canopy conductance (*g*_c_) to VPD (Law et al. 2001a). The soil water modifier (*f*_ϴ_) is calculated using the moisture ratio of current : available water and a soil water constant (*c*_ϴ_) and power (*r*_ϴ_) that reflect different soil types (Landsberg et al. 2003). For sandy loam at our study site, *c*_ϴ_ is 0.4 and *r*_ϴ_ is 7. The age modifier (*f*_age_) accounts for reductions in hydraulic and stomatal conductance as stands age.

The 3‐PG model the ratio of NPP to GPP is fixed at 0.47 (Waring et al. 1998). NPP is allocated to foliage, woody tissue, and root biomass pools based on species‐specific partitioning algorithms, which also depend on site and growth conditions, litterfall, and root turnover (Waring et al. 1998). The model uses a simple relationship to determine root growth and turnover to estimate belowground biomass allocation. Allometric ratios are used to determine the allocation of biomass to stems and foliage. Stem growth, stand density, and stem mortality are calculated according to the self‐thinning rule based on the negative relationship between tree density and stem mass (Landsberg and Waring 1997). Soil water balance is based on rainfall, irrigation, evapotranspiration, and runoff/drainage. Evapotranspiration (ET, J·m^−2^·s^−1^) is determined from the Penman‐Monteith equation and canopy conductance (Penman 1948, Monteith 1965, Monteith and Unsworth 2007):

(4) $$\text{ET}=\frac{e_{20}R_{n}+\left(\rho \lambda g_{\mathrm{bl}}\left(e_{s}-e_{a}\right)\right)}{\left(1+e_{20}+\frac{g_{\mathrm{bl}}}{g_{c}}\right)}$$

where *e*_20_ is the ratio of the slope of the saturation‐vapor pressure curve at *T*_air _= 20°C to the psychrometric constant (2.2); *R*_n_ is the net radiation (W/m^2^); ρ is density of air (1.2 kg/m^3^); λ is the latent heat of vaporization of water (2,460,000 J/kg); *g*_bl_ is boundary layer conductance, and *g*_c_ is canopy conductance (both in m/s); and *e*_s_ − *e*_a_ is the saturation vapor pressure deficit. *E* was converted to mol·m^−2^·s^−1^ to be used in the Peclet calculation in the δ^18^O submodel.

We updated the calculation of canopy conductance (*g*_c_) by multiplying it by the frost modifier (*f*_F_) to prevent any *E* from occurring on days with frost. *g*_c_ was calculated as:

(5) $$g_{c}=\left(TK_{2}+TK_{3}T_{\mathrm{av}}\right)g_{\mathrm{cmax}}\;f_{F}f_{\mathrm{age}}\left(\text{LAI}/{\text{LAI}}_{g_{\mathrm{cx}}}\right)$$

where TK_2_ and TK_3_ are temperature modifiers (0.244, 0.0368, respectively, (Wei et al. 2014a), *g*_cmax_ is maximum canopy conductance, LAI is leaf area index, and LAI_*g*cx_ is the LAI required for a stand to reach its *g*_cmax_ (3.3 m^2^/m^2^). The impact of this update is presented in Appendix S1: Fig. S2.

### Allometric equation to estimate stem biomass

Diameter at breast height (DBH) and biomass measured in *Pinus* species (Gholz et al. 1979) were used to determine the stem constant (*S*_c_) and stem power (*S*_p_) used in 3‐PG in (Wei et al. 2014b). Live branch mass, stem wood mass, and stem bark mass (Gholz et al. 1979) were summed to calculate total stem biomass. Total stem biomass was then plotted against stem DBH. The relationship between DBH and total biomass (*W*) was described by an exponential function:

(6) $$W=S_{c}{\text{DBH}}^{S_{p}}$$

where *S*_c_ = 0.0273 and *S*_p_ = 2.6405.

### Basal area increment calculation

To compare with observed BAI (cm^2^·tree^−1^·yr^−1^), simulated BAI (cm^2^·tree^−1^·yr^−1^) was calculated from modeled outputs of basal area and stand density as follows:

(7) $$\begin{array}{cc} \text{BAI}= & {\left[\frac{\text{basal area}(m^{2}/\text{ha})}{\text{stand density}(\text{trees/ha})}\times 10,000\frac{{\text{cm}}^{2}}{m^{2}}\right]}_{{\mathrm{year}}_{n}}\\ & -{\left[\frac{\text{basal area}(m^{2}/\text{ha})}{\text{stand density}(\text{trees/ha})}\times 10,000\frac{{\text{cm}}^{2}}{m^{2}}\right]}_{{\mathrm{year}}_{n-1}} \end{array}$$

where *n* represents a given year.

### δ^13^C_cell_ theory and submodel

The δ^13^C of photosynthate (δ^13^C_plant_) is described in Farquhar et al. (1982) as:

(8) $$\delta^{13}C_{\mathrm{plant}}\approx \delta^{13}C_{\mathrm{air}}-a-\left(b-a\right)\frac{c_{i}}{c_{a}}$$

where *a* is the kinetic fractionation effect associated with diffusion of CO_2_ through stomata (4.4‰), *b* is the net kinetic fractionation effect (27‰) associated with discrimination against ^13^C by the enzyme RUBISCO (ribulose bisphosphate carboxylase‐oxygenase) during C fixation, and *c*_i_/*c*_a_ is the weighted mean ratio of the intercellular CO_2_ concentration (*c*_i_) to that in the ambient air (*c*_a_; Farquhar et al. 1982, 1989). The *c*_i_ can be estimated from *c*_a_, photosynthesis (*A*), and canopy conductance (*g*_c_; Farquhar and Sharkey 1982):

(9) $$c_{i}=c_{a}-\frac{A}{0.66g_{c}}.$$

The value of 0.66 describes the ratio of diffusivities of CO_2_ to water vapor in air (Wei et al. 2014a). Therefore, tree‐ring δ^13^C_plant_ reflects factors that influence discrimination against ^13^C during photosynthetic CO_2_ fixation. These factors include the biochemical capacity to fix CO_2_ (*A*), and the conductance (*g*) to CO_2_ from the atmosphere to the sites of carboxylation. Although leaf‐level *g* includes stomatal conductance (*g*_s_) and mesophyll conductance, we assume the simplified equation from Farquhar et al. (1982, 1989), assuming that canopy conductance (*g*_c_) and *c*_i_ are sufficient to model δ^13^C_plant_ (Cernusak et al. 2003).

The δ^13^C submodel developed previously for 3‐PG (Wei et al. 2014a) treats the canopy as a big leaf (Farquhar et al. 1989) and combines Eq. (8) and Eq. (9) so δ^13^C_plant_ is calculated as

(10) $$\delta^{13}C_{\mathrm{plant}}\approx \delta^{13}C_{\mathrm{air}}-a-\left(b-a\right)\left(1-\frac{A}{c_{a}0.66g}\right).$$

To convert δ^13^C_plant_ of new photosynthate to δ^13^C of tree‐ring wood (δ^13^C_wood_), a constant offset (ε_sp_) of 1.99‰ was assumed (Wei et al. 2014a), similar to that observed in other *Pinus* species (Gessler et al. 2009, Wei et al. 2014b):

(11) $$\delta^{13}C_{\mathrm{wood}}=\delta^{13}C_{\mathrm{plant}}+\varepsilon_{\mathrm{sp}}.$$

This model was modified to include a constant offset (ε_wc_) of 1.5‰ observed in *P. ponderosa* (English et al. 2011) between the δ^13^C_wood_ and the δ^13^C of tree‐ring cellulose (δ^13^C_cell_):

(12) $$\delta^{13}C_{\mathrm{cell}}=\delta^{13}C_{\mathrm{wood}}+\varepsilon_{\mathrm{wc}}.$$

### δ^18^O_cell_ theory and submodel

The δ^18^O of plant tissue incorporates signals imparted by the δ^18^O values of source water (δ^18^O_s_) and water from the site of evaporation within the leaf (δ^18^O_es_), the latter of which is influenced by ^18^O‐evaporative enrichment from *E*, and invasion of isotopically depleted vapor (δ^18^O_v_), which is governed by the leaf relative humidity (Craig and Gordon 1965, Dongmann et al. 1974). Under steady state conditions

(13) $$\delta^{18}O_{\mathrm{es}}=\delta^{18}O_{s}+\varepsilon^{*}+\varepsilon_{k}+\left(\delta^{18}O_{v}-\delta^{18}O_{s}-\varepsilon_{k}\right)\frac{e_{a}}{e_{i}}$$

where δ^18^O_es_, δ^18^O_s_, and δ^18^O_v_ represent the oxygen isotopic composition of leaf water at the site of evaporation, source water, and atmospheric water vapor, respectively. *e*_a_/*e*_i_ is the ratio of ambient to saturation vapor pressure within the leaf, ε* is the equilibrium fractionation between liquid water and vapor, and ε_k_ is the kinetic fractionation factor of vapor diffusion from the leaf to the atmosphere; δ^18^O_v_ was estimated using *T*_av_ and was based on the assumption that vapor is in isotopic equilibrium with source water (Majoube 1971).

We estimated δ^18^O_s_ three different ways. First, since the Metolius River is spring fed with a long residence time (Manga 2001), we used a constant δ^18^O_s_ value over time obtained from measured δ^18^O of stem and river water samples (−14.2‰, Table 2). Second, we used monthly δ^18^O_s_ estimates for our site from Waterisotopes.org. Finally, because temperature and precipitation influence δ^18^O_s_, we developed a multiple linear regression model to estimate mean monthly δ^18^O_s_ at our site (Yang et al. 2011). To develop the model, we used precipitation δ^18^O measured weekly when precipitation occurred in Corvallis, Oregon from 2002 to 2017 (~500 observations) and temperature and precipitation obtained from PRISM. Corvallis was the closest location to our study site that had multiple years of measured precipitation δ^18^O that could be used as a proxy for δ^18^O_s_. We used BIC values to determine the model of best fit. The selected model equation was: δ^18^O_s_ = (−0.0347 × precipitation) + (0.154 × *T*_av_) + (−8.67) (*R* ^2^ = 0.24). Because the Metolius study site is east of Corvallis, Metolius δ^18^O_s_ values are naturally more negative than those of Corvallis. Therefore, we adjusted the model for our study site by adjusting the intercept of the multiple linear regression model to −11.57‰ to reflect the average annual difference in δ^18^O_s_ between sites (2.9‰) estimated from Waterisotopes.org. We then used PRISM precipitation and temperature values for our study site in the multiple linear regression model to estimate monthly δ^18^O_s_ at our study site. Monthly δ^18^O_s_ values from all three methods were then precipitation‐weighted based on water year (October–September) precipitation that accumulated up through the current month. For example, May δ^18^O_s_ would be weighted by the October‐May precipitation amount. We compared the results of all three methods (Appendix S1: Table S2, Fig. S3) and selected the δ^18^O_s_ that resulted in the greatest Pearson correlation coefficient (*R*) between modeled and observed δ^18^O_cell_ for both upland and riparian sites. Based on the *R* comparisons, the δ^18^O_s_ calculated from the multiple linear regression model was selected for both the upland and riparian sites and was used for the presented results (Appendix S1: Table S2, Fig. S3).

**Table 2:** δ^18^O of source river water, stem water, and atmospheric water vapor at the upland and riparian sites in 2002 and 2004

Location, sample type, and date	δ^18^O (‰)
Metolius River	
River water	
29 August 2002	−13.9
13 July 2004	−14.2
Upland	
Stem water	
29 August 2002	−13.3 ± 0.1
13 July 2004	−14.6
Water vapor	
13 July 2004	−26.0
Riparian	
Stem water	
13 July 2004	−14.2
Water vapor	
13 July 2004	−25.3

*Notes: N* = 1–4. Error shown is ±SE.

Leaf water δ^18^O (δ^18^O_lw_) heterogeneity can be explained further by the Peclet effect, which describes the ratio between the *E*‐induced mass flow (advection) of unenriched source water to the evaporative sites and the back diffusion of isotopically enriched water from the sites of evaporation (Farquhar and Lloyd 1993, Barbour 2007):

(14) $$\delta^{18}O_{\mathrm{lw}}=\delta^{18}O_{\mathrm{es}}\frac{\left(1-e^{-P}\right)}{P}$$

(15) $$P=\frac{\text{EL}}{\text{CD}}$$

where δ^18^O_lw_ is the steady state isotopic enrichment of mean leaf lamina water, P is the Peclet number describing the ratio of advection to diffusion, *E* is the leaf transpiration rate (mol·m^−2^·s^−1^), *L* is the scaled effective path length (m) for water movement from the veins to the site of evaporation, *C* is the molar density of water (55.56 × 10^3^ mol/m^3^), and *D* is the diffusivity of the heavy water isotopologue (H_2_^18^O) in water (2.66 × 10^−9^ m^2^/s). *L* is defined as the product of two components: *l*, the actual distance of the water pathway from xylem to the evaporative surface, and *k,* a scaling factor that accounts for the tortuosity of the path of water through a porous medium (Farquhar and Lloyd 1993, Barbour et al. 2000).

Isotopic fractionation during the incorporation of the δ^18^O_lw_ signal into cellulose of plant tissue is described by the following equation (Roden et al. 2000):

(16) $$\delta^{18}O_{\mathrm{cell}}=f_{O}\left(\delta^{18}O_{s}+\varepsilon_{o}\right)+\left(1-f_{O}\right)\left(\delta^{18}O_{\mathrm{lw}}+\varepsilon_{o}\right)$$

where *f*_O_ is the proportion of oxygen atoms that exchange with source water during cellulose formation (0.42; Roden et al. 2000), and ε_o_ is a fractionation factor of +27‰ associated with the water/carbonyl interactions (Yakir et al. 1990). The δ^18^O submodel in 3‐PG calculates δ^18^O_cell_ with and without the Peclet effect (i.e., substituting δ^18^O_es_ for δ^18^O_lw_ in Eq. (16)). The model was modified so the monthly modeled *E* is used in the Peclet calculation rather than a fixed *E*.

### Parameterization and calibration

We added the δ^18^O submodel in this study to the Wei et al. (2014a) version of 3‐PG with a δ^13^C submodel (model runs in Python version 2.7; Wei et al. 2018). Data S1 includes the Python version of the 3‐PG model with the δ^13^C and δ^18^O submodels. First, parameters were set to defaults used in previous applications of 3‐PG at this Metolius site (Appendix S1: Table S1). Next, several parameters were calibrated following the approach of (Wei et al. 2014a) and (Landsberg et al. 2003). Maximum canopy conductance (*g*_cmax_) and the coefficient describing the sensitivity of canopy conductance to VPD (*k*_g_) were calibrated based on previously reported values of *E* and *g* for *P. ponderosa* at this site (Law et al. 2000, 2001a), and the equation describing the relationship between *g* and VPD (Law et al. 2001a). Fertility rating (FR), foliage : stem partitioning ratio of tree diameter of 20 cm (pfs20), maximum root partitioning (*p*_rx_), maximum tree stem mass likely in mature stands of 1,000 trees/ha (wSx1000), and maximum quantum efficiency (α_cx_) were calibrated based on observed and previously reported values of LAI, basal area, BAI, stand density, δ^13^C_cell_, and δ^18^O_cell_ with other parameters held constant (Wei et al. 2014a). *L* was calibrated to match modeled and observed δ^18^O_cell_ with a Peclet effect included.

The measured trajectories of δ^13^C_cell_ and δ^18^O_cell_ at the upland site were prioritized for calibration over previously reported stand characteristics, because we had 107 yr of measured tree‐ring data; although the previously reported stand characteristics (Table 1) were measured at the same site, they were only for single years and not necessarily on the same trees for which we had the long‐term measured tree‐ring trajectories of δ^13^C_cell_, δ^18^O_cell_, and BAI. To do this, calibration of parameters as described above was determined first based on minimizing the root mean squared error (RMSE) between the measured and modeled values of δ^13^C_cell_, δ^18^O_cell_, and then BAI, and then previously reported stand characteristics.

The model trained on the upland site was then tested on the riparian site. Because the upland and riparian sites were <5 km apart, climate driving inputs and stand initialization values remained the same as those for the upland site, and the model was also run for the same time period as the upland site (i.e., from 1895 to 2002). Similar to the upland site, we compared the results of all three methods used to estimate monthly δ^18^O_s_ for the riparian site (Appendix S1: Table S2, Fig. S3) and selected the δ^18^O_s_ that resulted in the greatest *R* between modeled and observed δ^18^O_cell_. The trained model did not predict the observed trajectories of BAI and δ^13^C_cell_ at the riparian site. Thus, we adjusted maximum available soil water (ASW), maximum canopy conductance (*g*_cmax_), and wSx1000 to minimize the RMSE between the measured and simulated BAI, δ^13^C_cell_, and δ^18^O_cell_ of riparian trees. By adjusting these parameters, we demonstrated how model parameterization can be used to identify site and physiological differences between upland and riparian trees. Other parameters that increased the RMSE between the measured and simulated BAI, δ^13^C_cell_, and δ^18^O_cell_ of riparian trees were not adjusted.

### Sensitivity analysis

We investigated the sensitivity of modeled BAI, δ^13^C_cell_, δ^18^O_cell_ with Peclet, δ^18^O_es_, *E*,* g*_c_, GPP, and LAI to ±20% and ±40% changes in the following parameters: α_cx_, FR, *g*_cmax_, *k*_g_, maximum ASW, pfs20, *p*_rx_, and wSx1000. These parameters were selected because they are known to influence δ^13^C_cell_ and biomass allocation (Wei et al. 2014a), but it is unknown how they might influence δ^18^O_cell_. *L* was adjusted to evaluate the sensitivity of δ^18^O_cell_ with Peclet to changes in *L*. To conduct the sensitivity analysis, one parameter at a time was adjusted ±20% and ±40% of the original value while all other parameters were held constant. The % change in the aforementioned output response (BAI, δ^13^C_cell_, δ^18^O_cell_ with Peclet, δ^18^O_es_, *E*,* g*_c_, GPP, and LAI) was then quantified from the original output value. These % changes were averaged over the study period of 1895–2002. An output was considered “sensitive” if a change in parameter resulted in a ≥10% change in output.

### Statistics

Modeled and observed values were compared using simple linear regression, Pearson correlation coefficient (*R*), the coefficient of determination (*r* ^2^), and root mean squared error (RMSE) in SigmaPlot 13.0 (Systat Software, San Jose, California, USA). A one‐sample *t* test was used to test if mean difference for each year spanning 1895–2002 between observed and modeled BAI, δ^13^C_cell_, and δ^18^O_cell_, between upland and riparian sites, and between δ^18^O_cell_ with and without the Peclet effect was significantly different from zero.

## Results

**Figure 1:**
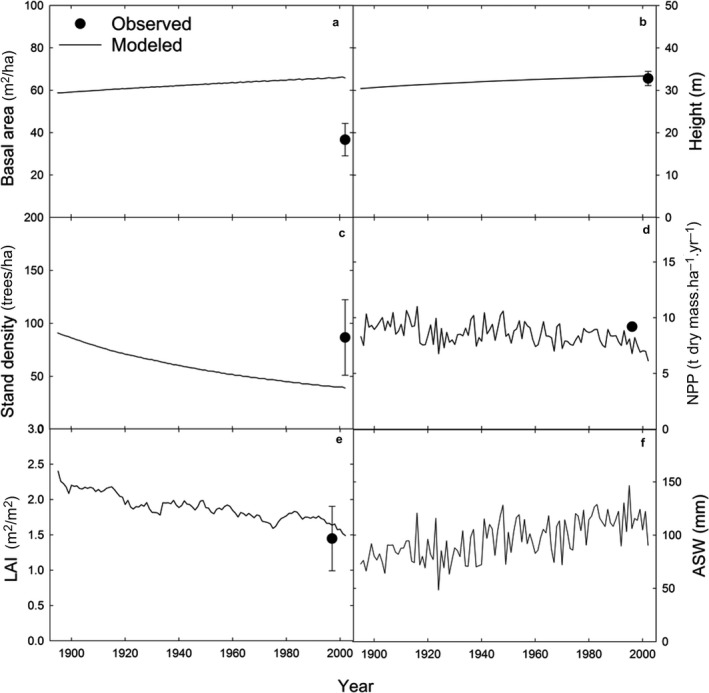
Modeled and observed (a) basal area, (b) height, (c) stand density, (d) net primary productivity (NPP), (e) leaf area index (LAI), and (f) available soil water (ASW) for 1895–2002 at the upland site. Observed data points represent means of previously reported values listed in Table 1. Error bars are ±SE.

**Figure 2:**
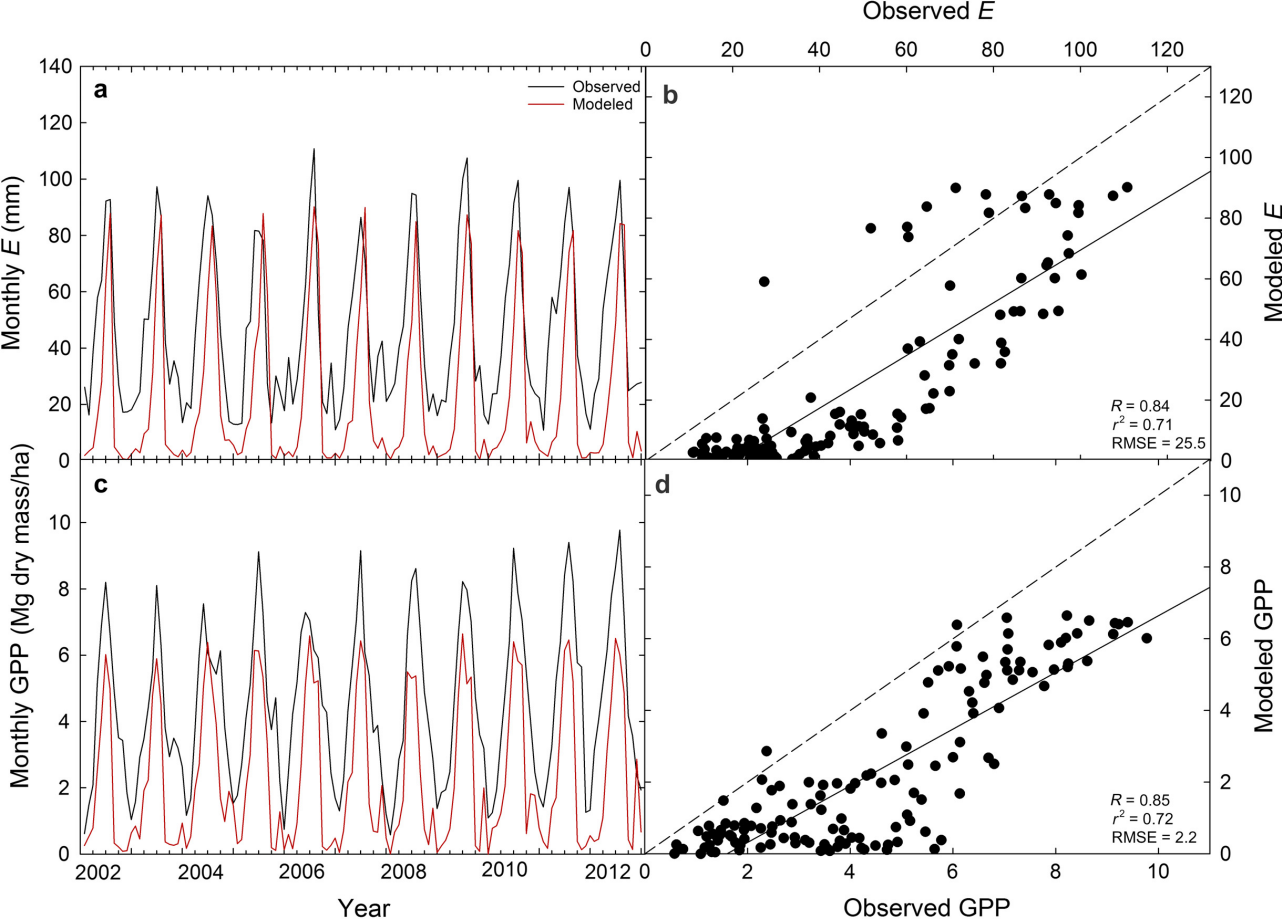
(a, c) Modeled and observed monthly transpiration (*E*, mm/month) and gross primary productivity (GPP) for 2001–2012 at the upland site and (b, d) observed vs. modeled *E* and GPP with model performance metrics: Pearson correlation coefficient (*R*), coefficient of determination (*r* ^2^), and root mean squared error (RMSE). The dashed line represents the 1:1 line.

**Figure 3:**
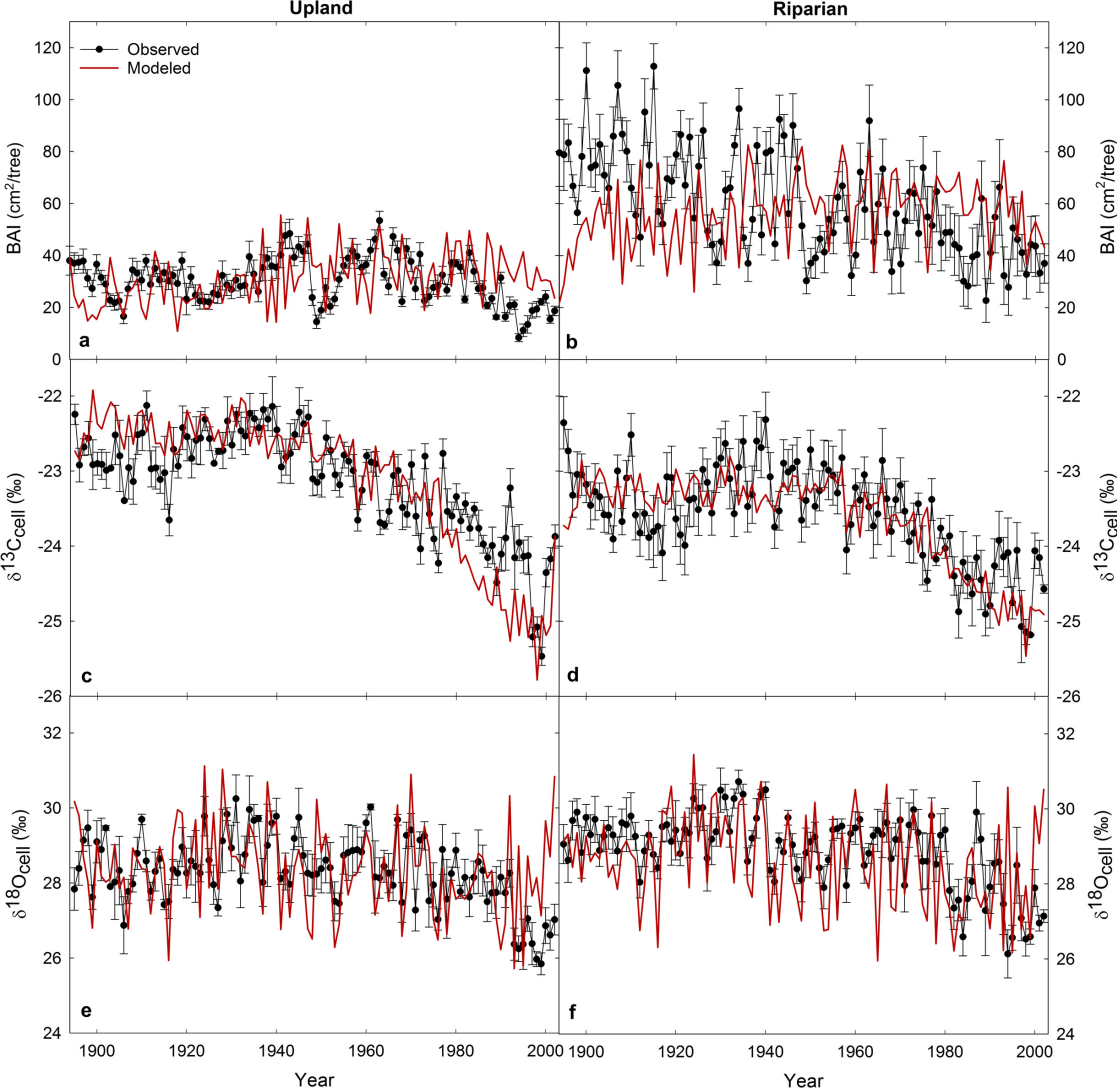
Modeled and observed time courses of basal area increment (BAI), δ^13^C_cell_, and δ^18^O_cell_ with the Peclet effect for (a, c, e) upland and (b, d, f) riparian trees. *N* = 5. Error bars are ±SE.

**Figure 4:**
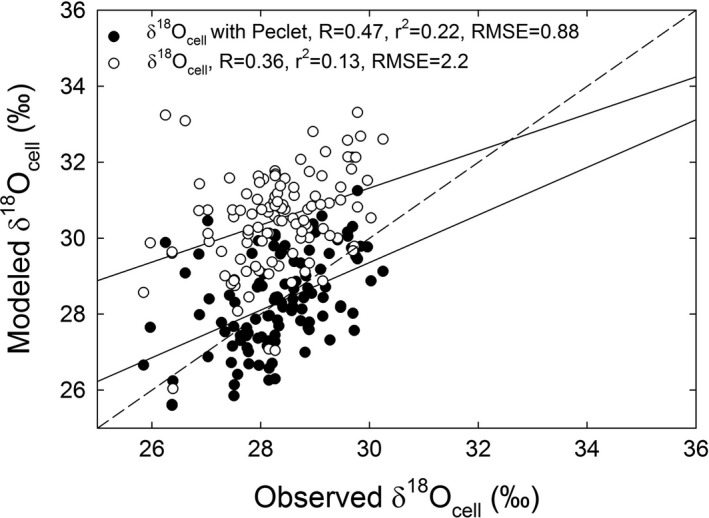
Observed vs. modeled values of δ^18^O_cell_ with and without the Peclet effect at the upland site for 1895–2002 with model performance metrics: Pearson correlation coefficient (*R*), coefficient of determination (*r* ^2^), and root mean squared error (RMSE). The dashed line represents the 1:1 line.

Parameterizing 3‐PG by prioritizing fit to measured BAI, δ^13^C_cell_, and δ^18^O_cell_, and secondarily previously reported stand characteristics, allowed the model to predict stand characteristics at the upland site reasonably well (Figs. 1, 2). Predicted BAI was within range of observed BAI values (Fig. 3a) where the mean difference between observed and modeled BAI for each year spanning 1895–2002 was not significantly different from zero (*P* = 0.67; observed BAI = 30.3 ± 0.9 cm^2^/yr, modeled BAI = 30.8 ± 1.0 cm^2^/yr). The model also predicted δ^13^C_cell_ within range of observed δ^13^C_cell_ at the upland site (Fig. 3c) where the mean difference between observed and modeled δ^13^C_cell_ for each year spanning 1895–2002 was not significantly different from zero (*P* = 0.85; observed δ^13^C_cell_ = −23.1‰ ± 0.07‰, modeled δ^13^C_cell_ −23.1‰ ± 0.09‰). Predicted δ^18^O_cell_ with Peclet was also within range of the upland site (Fig. 3e) where the mean difference between observed and modeled δ^18^O_cell_ for each year spanning 1895–2002 was not significantly different from zero (*P* = 0.94; observed δ^18^O_cell_ = 28.3 ± 0.1‰, modeled δ^18^O_cell _= 28.9‰ ± 0.01‰). The Peclet effect improved the correlation between observed and modeled δ^18^O_cell_ (Fig. 4; Appendix S1: Table S2) while δ^18^O_cell_ without the Peclet effect significantly overestimated observed δ^18^O_cell_ (*P* ≪ 0.001).

Compared to upland trees, observed BAI of riparian trees was consistently and significantly greater (Fig. 3a,b) where the 1895–2002 mean observed BAI of riparian trees was 60.0 ± 1.9 cm^2^/yr, nearly twice that of upland trees, and the mean difference between upland and riparian observed BAI for each year spanning 1895–2002 was significantly different from zero (*P* < 0.001). Riparian trees also exhibited consistently and significantly lower (more negative) mean observed δ^13^C_cell_ than upland trees (Fig. 3c, d); the 1895–2002 mean observed δ^13^C_cell_ of riparian trees was −23.8 ± 0.04‰ which was 0.7‰ more negative than upland trees, and the mean difference between upland and riparian observed δ^13^C_cell_ for each year spanning 1895–2002 was significantly different from zero (*P* < 0.001). The observed δ^13^C_cell_ of the upland trees ranged from −24.8‰ to −22.3‰, while observed δ^13^C_cell_ of the riparian trees ranged from −25.3‰ to −23.1‰. In contrast to BAI and δ^13^C_cell_, the mean difference between upland and riparian observed δ^18^O_cell_ for each year spanning 1895–2002 was not significantly different from zero (*P* = 0.16; upland δ^18^O_cell_ = 28.3 ± 0.1‰, riparian δ^18^O_cell_ = 28.9 ± 0.1‰, Fig. 3e, f). The observed δ^18^O of river water was similar to the observed δ^18^O of stem water in both upland or riparian trees (Table 2). The observed δ^18^O of stem water and atmospheric water vapor were also similar between upland and riparian trees (Table 2). Because observed δ^18^O data for river water, stem water, and water vapor were only available for one value per sampling date, we could not test for statistically significant differences.

The model trained on the upland site did not predict the observed BAI and δ^13^C_cell_ trajectories of riparian trees given their significantly greater BAI and lower δ^13^C_cell_ trajectories compared to upland trees (Fig. 3). However, we hypothesized that riparian trees had access to a greater water supply, which would increase canopy conductance and thus influence BAI and δ^13^C_cell_. To test this hypothesis, we increased maximum available soil water (ASW) from 163 to 300 mm, maximum canopy conductance (*g*_cmax_) from 0.014 to 0.0145 m/s, and decreased the maximum tree stem mass likely in mature stands of 1,000 trees/ha(wSx1000) from 110 to 100 kg/tree. These parameter changes for the riparian trees reproduced the greater BAI and lower δ^13^C_cell_ values observed in riparian trees compared to upland trees without substantially influencing the modeled δ^18^O_cell_ of riparian trees (Fig. 3). The mean differences between riparian observed and modeled BAI, δ^13^C_cell_, and δ^18^O_cell_ for each year spanning 1895–2002 were not significantly different from zero (*P* = 0.11, 0.20, 0.12, respectively; Fig. 3). By updating maximum ASW, *g*_cmax_, and wSx1000, we demonstrate how model parameterization can be used to identify site and physiological differences between upland and riparian trees.

**Figure 5:**
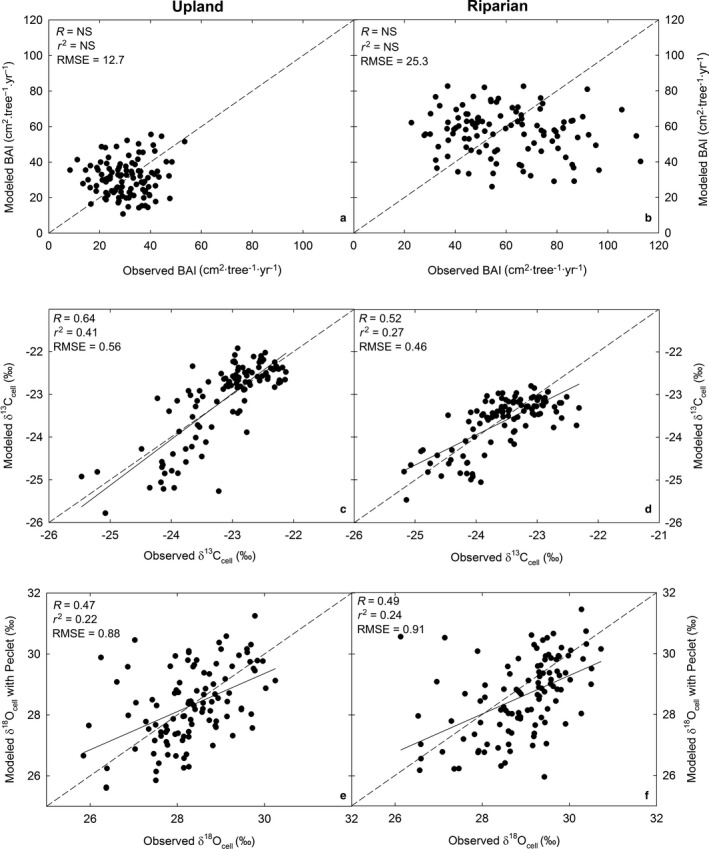
Modeled vs. observed values of (a, b) BAI, (c, d) δ^13^C_cell_, and (e, f) δ^18^O_cell_ with Peclet at the upland and riparian sites for 1895–2002 with model performance metrics: Pearson correlation coefficient (*R*), coefficient of determination (*r* ^2^), and root mean squared error (RMSE). The dashed line represents the 1:1 line.

Although the mean differences between modeled and observed BAI for 1895–2002 at both the upland and riparian sites were not significantly different from zero (*P* > 0.05), modeled and observed BAI were not significantly correlated (simple linear regression, *P* > 0.05) and RMSE was 12.7 and 25.3, respectively (Fig. 5). In contrast, modeled and observed δ^13^C_cell_ at both the upland and riparian sites were significantly correlated (simple linear regression, *P* < 0.001, *R* = 0.64, 0.52, *r* ^2^ = 0.41, 0.27, respectively) and RMSE was 0.56 and 0.46, respectively (Fig. 5). Modeled and observed δ^18^O_cell_ with Peclet at both the upland and riparian sites were also significantly correlated (simple linear regression, *P* < 0.001, *R* = 0.47, 0.49, *r* ^2^ = 0.22, 0.24, respectively) and RMSE was 0.88 and 0.91, respectively (Fig. 5).

**Figure 6:**
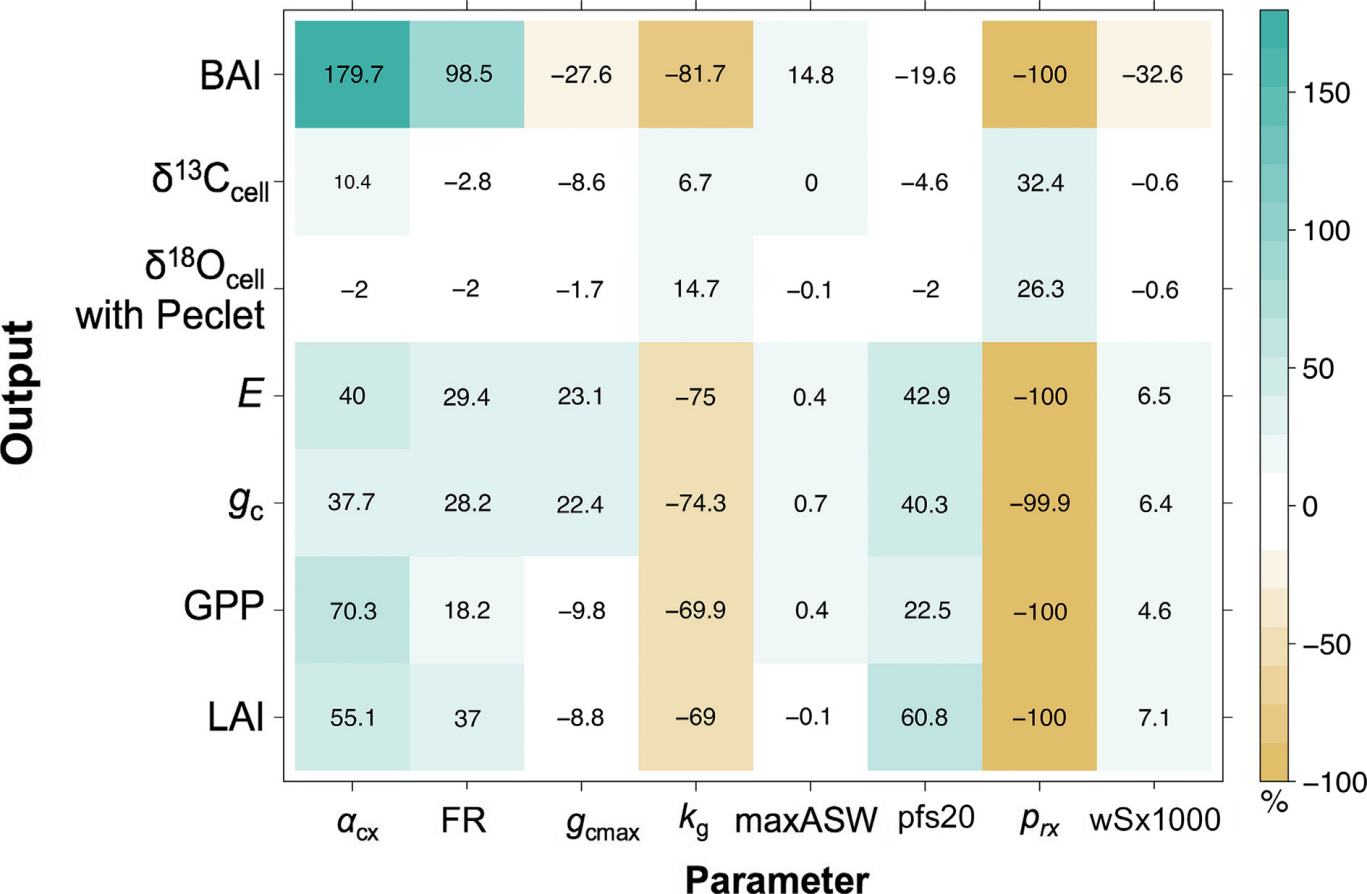
Sensitivity analysis results of the effect of a 40% increase in parameters: maximum quantum efficiency (α_cx_), fertility rating (FR), maximum canopy conductance (*g*_cmax_), sensitivity of canopy conductance to VPD (*k*_g_), maximum available soil water (maxASW), foliage : stem partitioning ratio for tree diameter of 20 cm (pfs20), maximum root partitioning (*p*_rx_), and maximum tree stem mass in mature stands of 1,000 trees/ha (wSx1000) on the percent change in output variables: basal area increment (BAI), δ^13^C_cell_, δ^18^O_cell_ with Peclet, transpiration (*E*), canopy conductance (*g*_c_), gross primary productivity (GPP), and leaf area index (LAI). Appendix S1: Table S3 shows all sensitivity analysis values including the effect of ±20% and ±40% shifts in parameters on percent change in output variables.

The sensitivity analysis showed that BAI, *E*,* g*_c_, GPP, and LAI were sensitive (i.e., each variable was changed by ≥10% of its original value) to changes in each of the tested parameters: α_cx_, FR, *k*_g_, pfs20, and *p*_rx_ (Appendix S1: Table S3; Fig. 6). In contrast, δ^13^C_cell_ was only sensitive to changes in α_cx_, *g*_cmax_, and *p*_rx_. δ^18^O_es_ was not sensitive to any parameter, while δ^18^O_cell_ with Peclet was sensitive to α_cx_, FR, *k*_g_, pfs20, and *p*_rx_.

**Figure 7:**
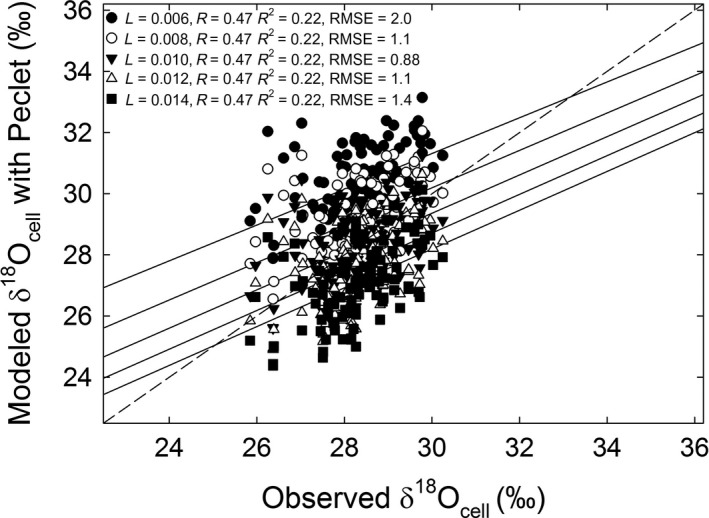
Modeled vs. observed δ^18^O_cell_ with Peclet for different values of *L*. *L* values are ±20% and ±40% of the optimized *L *=* *0.010 m.

*L* was estimated using δ^18^O_cell_ with Peclet, where an *L* value of 0.010 m resulted in modeled δ^18^O_cell_ that best predicted the observed δ^18^O_cell_ (Fig. 7).

## Discussion

We used long‐term tree‐ring growth, δ^13^C_cell_, and δ^18^O_cell_ to demonstrate for the first time the use of 3‐PG with the newly added δ^18^O submodel and to investigate physiological differences between old‐growth upland and riparian *P. ponderosa*. This application of 3‐PG in conjunction with measured BAI and the dual isotope approach across 107 yr is unprecedented. Model performance was better for both δ^13^C_cell_ and δ^18^O_cell_ than for BAI and stand growth characteristics (Figs. 1, 2, 3, 5). The δ^18^O submodel with the Peclet effect improved predictions of δ^18^O_cell_ (Fig. 4; Appendix S1: Table S2) because it incorporates 3‐PG's monthly *E* predictions, and also reflects growth and allocation processes, unlike previous δ^18^O_cell_ models based just on leaf water and cellulose isotope equations driven by precipitation and vapor isotopes and relative humidity. Our approach also provided a way to estimate *L* (Fig. 7), which is typically an unmeasurable component of the Peclet effect. By using the model to aid our understanding of the physiology driving the BAI, δ^13^C_cell_, and δ^18^O_cell_ trajectories at the upland and riparian sites, we propose that upland and riparian trees were using the same source of water isotopically, but that greater water availability at the riparian site increased tree growth and lowered δ^13^C_cell_ values.

### Model performance

Model parameterization based on previously reported parameters and stand characteristics for *P. ponderosa* resulted in overall reasonable values of stand characteristics with minor discrepancies (Fig. 1). Modeled height and LAI fell within the range of the mean of previously reported values. Modeled LAI reached ~2.2 m^2^/m at a stand age of 30 yr (not shown), consistent with observed values for *P. ponderosa* at that same age (Law et al. 2001b), indicating the LAI was well predicted beyond our study period of 1895–2002 when the trees ranged from 155 to 262 yr in age. NPP was within range of previously reported NPP for *P. ponderosa* at the Metolius site (Law et al. 2000). Modeled *E* and GPP were only slightly lower than the observed ecosystem water vapor flux (LE) and GPP AmeriFlux values (Fig. 2), but LE includes all forms of evaporation from the soil and understory, which is likely why observed LE was slightly greater than simulated *E* (i.e., transpiration only*)*.

Minor discrepancies existed between modeled and previously reported stand growth characteristics. These discrepancies arose due to our prioritizing model calibration first on measured ring width and isotope values, and secondarily on previously reported stand‐level values, which differed from our study trees. Previously reported stand densities ranged from 54 to 137 trees/ha (Table 1) and previously reported diameter at breast height (DBH) ranged from 55 to 63 cm (Law et al. 2001a, 2001b, Youngblood et al. 2004, Warren et al. 2005) at this site. However, the trees cored in this study had DBH values ranging 88–112 cm (Table 1), as we preferentially selected old and large dominant trees. Thus, the model predicted fewer, larger diameter trees (Fig. 1), as 3‐PG utilizes the established negative relationship between DBH and stand density (Meyer 1938). Because 3‐PG was developed for even‐aged stands, modeled basal area was greater than the basal area previously reported for younger or mixed‐aged stands at the site (Fig. 1a). However, discrepancies between modeled and previously reported stand density and basal area are not surprising, as we prioritized tree‐ring growth and isotope trajectories for model parameterization. This calibration method is consistent with Landsberg et al. (2003).

Although modeled BAI values were within range of observed values (Fig. 3a, b), the ability of 3‐PG to predict variation in BAI over time was sub‐optimal (i.e., larger RMSE; Fig. 5a, b). 3‐PG was developed to simulate even‐aged plantation stands, and thus stand growth is often challenging to model accurately in unmanaged forests (e.g., modeled DBH in Wei et al. 2014a). This is in part because microclimatic and site‐specific conditions such as non‐uniform tree spacing are not accounted for in a generalizable forest stand growth model like 3‐PG (Waring et al. 2016). This sub‐optimal predictive ability of the model is also likely due to the relatively long period (1895–2002, 107 yr) over which we applied the model compared to previous studies. Waring and Gao (2016) compared the tree‐ring index of *Picea crassifolia* to normalized 3‐PG‐predicted diameter growth for 52 yr. Wei et al. (2014a) compared observed and predicted DBH of *Abies grandis* for 50 yr with sub‐optimal success. The 107‐yr timeframe distinguishes this study from previous tree‐ring research using 3‐PG; however, it is likely that 3‐PG may more accurately predict observed growth across a shorter timeframe (Law et al. 2000, Wei et al. 2014b). These latter studies applied 3‐PG to *P. ponderosa* only for 25 and 2 yr, respectively. Although 3‐PG's sub‐optimal ability to predict BAI may potentially limit its capacity to model tree‐ring isotopes, the model still simulated δ^13^C_cell_ and δ^18^O_cell_ well in this study (i.e., small RMSE, Figs. 3, 5). This is likely because the model predicted monthly *E* and GPP relatively well (Fig. 2). This suggests that 3‐PG with the δ^13^C and δ^18^O submodels may be used to predict tree‐ring isotopes over long timeframes if the model can reasonably predict at least one metric of productivity (e.g. GPP, BAI, basal area, DBH, height).

### δ^18^O submodel

To our knowledge, this is the first time a δ^18^O_cell_ submodel has been added to 3‐PG. This first test of 3‐PG with a δ^18^O_cell_ submodel is unique because the *E* output is used in the calculation of the Peclet number and thus δ^18^O_cell_ with Peclet (Eqs. (14), (15), (16)), allowing this application of the δ^18^O_cell_ submodel to shed light on the mechanistic controls of δ^18^O_cell_. Both δ^13^C_cell_ and δ^18^O_cell_ were shown to be sensitive to fewer parameters than other model outputs like BAI, LAI, *g*_c_, and *E*. δ^13^C_cell_ can be used to constrain gas exchange parameters, including α_cx_ and *g*_cmax_, before allocation processes, as indicated by the sensitivity analysis (Fig. 6; Appendix S1: Table S3), and consistent with the findings of Wei et al. (2014a). In contrast, δ^18^O_cell_ is calculated downstream of other model calculations using the *E* output. Thus, δ^18^O_cell_ with Peclet was sensitive to parameters that also influence *E*. Since *E* and *g*_c_ both influence δ^13^C_cell_ and δ^18^O_cell_, model parameters that influence *E* and *g*_c_ would need to be adjusted to reasonably predict observed values of both δ^13^C_cell_ and δ^18^O_cell_. This further supports that 3‐PG with the δ^13^C and δ^18^O submodels may be used to predict tree‐ring isotopes if the model can reasonably predict *E* and GPP.

Similar to other studies, we found that incorporating the Peclet effect improved δ^18^O_cell_ estimates (Fig. 4; Appendix S1: Table S2) and that modeled δ^18^O_cell_ without a Peclet effect overestimated observed δ^18^O_cell_ (Barbour et al. 2000, Kahmen et al. 2008, Ripullone et al. 2008, Holloway‐Phillips et al. 2016). The δ^18^O_es_ and thus δ^18^O_cell_ without the Peclet effect were not sensitive to any of the tested parameters because both are calculated solely with δ^18^O_s_ and climate inputs (Eq. (13)). In contrast, δ^18^O_cell_ with Peclet was calculated using modeled *E*, demonstrating how including 3‐PG predictions of water use, growth, and allocation can improve predictions of δ^18^O_cell_. This was evident by the finding that predicted δ^18^O_cell_ with Peclet was more closely correlated with observed values than predicted δ^18^O_cell_ without the Peclet effect (Fig. 4; Appendix S1: Table S2). This also allowed *L*, the unmeasurable component of the Peclet effect, to be estimated (Fig. 7) and our estimate fell within the range of previously reported values for *Pinus* species (Song et al. 2013). This provides support for the use of a well‐parameterized model based on measured and previously reported stand characteristics to estimate *L* and evaluate the impacts of variation in leaf‐level physiology on the stand scale, although this needs to be tested further.

### Comparing upland and riparian tree physiology

The model helped to explain the physiological mechanisms underlying the significant differences in BAI and δ^13^C_cell_ between upland and riparian trees. We hypothesized that close proximity of the riparian trees to the Metolius River would increase water availability and reduce drought stress compared to the upland trees, thereby altering canopy conductance, BAI, and δ^13^C_cell_. By adjusting maximum ASW, *g*_cmax_, and wSx100 to better predict the greater BAI and lower δ^13^C_cell_ observed at the riparian site (Figs. 3, 5), we demonstrate how model parameterization can be used to investigate site and physiological differences between upland and riparian trees. This suggests that our hypothesis for the greater BAI and lower δ^13^C_cell_ was correct (Orwig and Abrams 1997, Adams and Kolb 2004). Because trees modulate stomatal conductance with water availability to maintain hydraulic function, the greater water availability also allowed riparian trees to maintain hydraulic function, gas exchange, and growth throughout more of the growing season (Panek and Goldstein 2001), consistent with the increase in *g*_cmax_ for riparian trees. This resulted in greater ^13^C discrimination due to lower relative stomatal constraints on *A* and thus greater CO_2_ supply, imparting a lower δ^13^C_cell_ signal in tree rings compared to upland trees (McCarroll and Loader 2004). Because *g*_cmax_ is related to hydraulic properties such as soil‐to‐leaf hydraulic conductance, greater *g*_cmax_ is consistent with greater sapwood‐specific native conductivity observed in *P. ponderosa* at a riparian site compared to an upland slope site (Stout and Sala 2003). The adjusted *g*_cmax_ value of 0.0145 m/s is within range for this species and site (Law et al. 2000, 2001a, Coops et al. 2005).

Given the greater measured growth and lower δ^13^C_cell_ of riparian trees and differing access to river water, we also expected δ^18^O of the riparian trees to differ from that of the upland trees due to differences in source water and/or in leaf‐level physiology. We had also expected that the δ^18^O of source water (δ^18^O_s_) would be constant over time for riparian trees (Manga 2001), and their δ^18^O_cell_ variance would only be related to climate (e.g., relative humidity, VPD) and leaf‐level physiology, while the δ^18^O_cell_ variance of the upland trees would be related to all three drivers. However, observed δ^18^O_cell_ did not substantially differ between the upland and riparian trees (Figs. 3, 5). To examine the mechanisms underlying the unexpectedly similar δ^18^O_cell_ trajectories between upland and riparian sets of trees, we discuss potential drivers of patterns in δ^18^O_cell_: relative humidity, leaf‐level physiology, and δ^18^O_s_ (Farquhar et al. 2007, Saugier et al. 2012) in terms of model performance and parameters.

First, differences in relative humidity and VPD can alter tree‐ring δ^18^O_cell_ (Kahmen et al. 2011, Voelker et al. 2014a) as shown by a significant correlation between VPD and δ^18^O_cell_ (Pearson correlation coefficient *R* = 0.20, *P* = 0.04, data not shown). However, we expected that the upland and riparian trees experienced similar evaporative demand because they were <5 km from each other. The similarity of the δ^18^O_cell_ time series and high correlation between upland and riparian δ^18^O_cell_ trajectories (*P* < 0.001, *R* = 0.73) also is consistent with this assumption.

Second, leaf‐level physiology may contribute to δ^18^O_cell_ patterns (Flanagan and Ehleringer 1991). The greater modeled *g*_cmax_, greater BAI, and lower δ^13^C_cell_ of the riparian trees suggested that riparian trees may have different leaf‐level gas exchange compared to the upland trees. However, δ^18^O_cell_ was not considered sensitive to changes in *g*_cmax_ in the model (Fig. 6; Appendix S1: Table S3) and unexpectedly the δ^18^O_cell_ trajectories did not differ between sets of trees.

Finally, δ^18^O_s_ is considered to be a primary driver of δ^18^O_cell_ (Treydte et al. 2014). The difference in proximity of the upland and riparian trees to the Metolius River suggested that they may use different sources of water (Marshall and Monserud 2006) where riparian trees may rely primarily on river water and upland trees may rely on both river water and precipitation (Stout and Sala 2003, Kerhoulas et al. 2013). This is why we expected that riparian trees would have a constant δ^18^O_s_ while upland trees would not. However, several lines of evidence suggest that upland and riparian trees were not using different sources of water: (1) observed δ^18^O_cell_ from the upland and riparian sites were both most correlated with modeled δ^18^O_cell_ with Peclet calculated using δ^18^O_s_ determined from the same method (multiple linear regression model, Appendix S1: Table S2); (2) the observed δ^18^O signal of the tree‐ring cellulose, stem water, and atmospheric water vapor were similar at both sites (Table 2); and (3) the δ^18^O_s_ from the Metolius River is reflected in the stem water of both upland and riparian trees (Table 2). Interestingly, both the upland and riparian sites displayed a decline in δ^18^O_cell_ in 1993–2002 that is likely related to site‐specific conditions, as this δ^18^O_cell_ decline was not observed in other central Oregon *P. ponderosa* (Roden and Ehleringer 2007), although they only measured latewood as compared to our whole‐ring measurements. To help identify what may be driving this anomalous decline in δ^18^O_cell_, we conducted a simple sensitivity analysis examining the effect of temperature, RH, *L*,* E*, and δ^18^O_s_ on δ^18^O_cell_. We found that a decrease in temperature, an increase in RH, an increase in *L*, an increase in *E*, and a decrease in δ^18^O_s_ can lower δ^18^O_cell_ (Appendix S1: Fig. S4). The δ^18^O_cell_ decline may have been caused by any combination of these factors unique to our study site. The presence of this δ^18^O_cell_ decline imprinted in the growth rings of trees from both sites provides more support that the upland and riparian sites and trees were likely using similar sources of water. However, the greater BAI, lower δ^13^C_cell_, and increased maximum ASW and *g*_cmax_ strongly suggested that the riparian trees had greater access to the same source of water (i.e., greater water availability) compared to upland trees.

## Conclusions

We tested the 3‐PG model with the δ^13^C_cell_ submodel and the newly added δ^18^O_cell_ submodel using long‐term trajectories of measured growth, δ^13^C_cell_, and δ^18^O_cell_ of old‐growth *P. ponderosa* in central Oregon. The unprecedented use of a 107‐yr period for which we had growth and isotope measurements revealed the model's strength in predicting δ^13^C_cell_ and δ^18^O_cell_ reasonably well across long timeframes but also highlighted the model's limitations in predicting certain stand growth characteristics. This first test of 3‐PG with a δ^18^O_cell_ submodel improves our understanding of mechanistic drivers of δ^18^O_cell_. Because δ^18^O_cell_ with the Peclet effect is calculated using stand‐level *E* output predicted by 3‐PG, the Peclet effect improved estimates of δ^18^O_cell_ and demonstrated that *L* and leaf‐level physiology may be estimated using a well‐parameterized model. The model helped to explain physiological drivers underlying the tree‐ring growth, δ^13^C_cell_, and δ^18^O_cell_ trajectories measured on the upland and riparian trees. The application of 3‐PG with the δ^13^C_cell_ and δ^18^O_cell_ submodels to the upland and riparian sets of trees indicates the potential of such coupled models to be parameterized for diverse stands using site‐ and stand‐specific information for examining the physiological mechanisms underlying forest responses to changes in climate.

## Note

1
http://oldprism.nacse.org/
