# Supplementary material for: Investigating old‐growth ponderosa pine physiology using tree‐rings, δ13C, δ18O, and a process‐based model
Source: Ecology. 2019 Apr 15;100(6):e02656. doi: 10.1002/ecy.2656 (PMC6645703; doi:10.1002/ecy.2656)
Supplement: Supplementary file 1 [file ECY-100-na-s001.pdf]

SUPPORTING INFORMATION

Table S1. Parameters for old-growth *Pinus ponderosa* at the Metolius site, Oregon. Default values from 3PGpjs vsn 2.7, 2010. Parameters were calibrated in the convention of (Wei et al. 2014a).

Parameter	Units	Abbreviation	Value	Reference
Initial conditions				
Initial foliage biomass	kg ha^{-1}	InitialWF	1.5	(Wei et al. 2014b)
Initial root biomass	kg ha^{-1}	InitialWR	1.4	(Wei et al. 2014b)
Initial stem biomass	kg ha^{-1}	InitialWS	0.9	(Wei et al. 2014b)
Initial stocking	trees ha^{-1}		550	(Warren et al. 2005)
Initial available soil water	mm	InitASW	30	Default
Maximum available soil water	mm	Maximum ASW	163	(Law et al. 2000)
Allometric relationships and partitioning				
Foliage:stem partitioning ratios for D=2 cm	-	*pfs2*	1.2745	Coops et al. (2005)
Foliage:stem partitioning ratios for D=20 cm	-	*pfs20*	0.77	Calibrated
Stem constant	-	S_c	0.0561	Equation 5
Stem Power	-	S_p	2.488	Equation 5
Maximum root biomass partitioning	-	p_{rx}	0.95	Calibrated
Minimum root biomass partitioning	-	p_{rn}	0.25	Default
Modifiers for photosynthesis and NPP/GPP				
Maximum temperature	°C	T_{max}	40	Law et al. (2000)
Minimum temperature	°C	T_{min}	-2	
Optimum temperature	°C	T_{opt}	20	Law et al. (2000)
Days production lost per frost day	days	k_F	1	Default
Assimilate use efficiency	-		0.47	Default
Soil water and fertility				
Soil type	-		Sandy loam	(Law et al. 2000)
Fertility rating	-	FR	0.1	Calibrated
Value of 'fN' when FR=0	-	fN_0	1	Default
Value of m when FR = 0	-	m_0	0	Default
Age modifier (f_{age})				
Maximum age	years	MaxAge	500	

Power of relative age in function for f_{age}	-	n_{Age}	4	Default
Relative age to five f_{age}=0.5	-	r_{Age}	0.95	Default
Litterfall and root turnover				
Maximum litterfall rate	month^{-1}	γ_{Fx}	0.021	Default
Coefficients in litterfall rate at t=0	month^{-1}	γ_F	0.001	Default
Age at which litterfall rate has median value	month	$t_{\gamma F}$	36	Default
Root turnover rate	month^{-1}	γ_R, Rttover	0.04	Default
Conductance				
Maximum canopy conductance	m s^{-1}	g_{cmax}	0.014	Calibrated
LAI required for maximum canopy conductance	-	LAI_{gcx}	3.3	Default
Response of canopy conductance to VPD	mbar^{-1}	k_g	0.05	Calibrated
Canopy boundary layer conductance, assumed constant	m s^{-1}	BL_{cond}	0.2	(Law et al. 2000)
Stem mortality				
Maximum stem mass (kg) likely in mature stands of 1000 trees ha^{-1}	kg tree^{-1}	*wSx1000*	110	Calibrated
Power in self-thinning law	-	thinPower	1.5	Default
Leaf mortality fraction	-	m_F	0.0	Default
Root mortality fraction	-	m_R	0.2	Default
Stem mortality fraction	-	m_S	0.2	Default
Branch & bark fraction at age 0	-	$frac_{BB0}$	0.15	Default
Branch & bark fraction for mature trees	-	$frac_{BB1}$	0.15	Default
Stand age for fracBB = (fracBB0+fracBB1)/2	years	t_{BB}	1.5	Default
Canopy structure and processes				
Specific leaf area for mature trees (m^2/kg)	m^2 kg^{-1}	SLA_1	4.2	(Law et al. 2000)
Stand age (years) for SLA = (SLA0+SLA1)/2	m^2 kg^{-1}	t_{SLA}	2.5	(Law et al. 2000)
Age at full canopy cover (years)	years	fullCanAge	15	Default
Radiation extinction coefficient	-	k	0.5	Default
LAI required for maximum rainfall interception	-	LAImaxIntcptn	5	Default
Max proportion of rainfall intercepted by canopy	-	MaxIntcptn	0.1	Default
Canopy quantum efficiency	mol C (mol PAR)$^{-1}$	α_{cx}	0.04	Calibrated

Power term used for describe the trajectory of canopy closure	-	CanPower	1	Default
Added parameter for tree height	-	HtC_0	4.85	Calibrated
Added parameter for tree height	-	HtC_1	-7.0	Default
Basic wood density	t m^{-3}		0.4	Default
δ¹³C submodel				
Conductance CO2 to water	-	RGcGW	0.66	Default
$\delta^{13}C$ difference of modeled tissue and new photosynthate	‰	δ^{13}CTissueDif	1.7	(Wei et al. 2014a)
Fractionation against ^{13}C in diffusion through air	‰	aFracDiffu	4.4	(Farquhar and Sharkey 1982)
Enzymatic fractionation by Rubisco	‰	bFracRubi	27	(Farquhar and Sharkey 1982)
Temperature modifier for g_c:k_2	-	TK_2	0.244	(Wei et al. 2014a)
Temperature modifier for g_c:k_3	-	TK_3	0.0368	(Wei et al. 2014a)

Table S2. Pearson correlation coefficients (R) between observed upland and riparian $\delta^{18}O_{cell}$ and 3-PG-modeled $\delta^{18}O_{cell}$ with and without the Peclet effect using: $\delta^{18}O_s$ calculated from a multiple linear regression model (linear model $\delta^{18}O_s$), $\delta^{18}O_s$ obtained from waterisotopes.org (Waterisotopes.org $\delta^{18}O_s$), and a constant $\delta^{18}O_s$ value based on measured $\delta^{18}O_s$ of stem water (constant $\delta^{18}O_s$). All coefficients were significant at $P<0.01$.

		**Modeled $\delta^{18}O_{cell}$ with Peclet**			**Modeled $\delta^{18}O_{cell}$ without Peclet**		
		Linear model $\delta^{18}O_s$	*Waterisotopes.org $\delta^{18}O_s$*	*Constant $\delta^{18}O_s$*	*Linear model $\delta^{18}O_s$*	*Waterisotopes.org $\delta^{18}O_s$*	*Constant $\delta^{18}O_s$*
**Observed $\delta^{18}O_{cell}$**	*Upland*	0.46	0.44	0.45	0.36	0.35	0.35
	Riparian	0.49	0.45	0.45	0.37	0.28	0.32

Table S3. Sensitivity analysis evaluating the effect of ±20% and ±40% changes in parameters on % change in outputs. Bolded outputs are those that changed ≥10%, are considered 'sensitive.'

Parameter	*Output*	*-40%*	*-20%*	*+20%*	*+40%*
α_{cx}	**BAI**	-98.4	-69.6	83.3	179.7
	**$\delta^{13}C_{cell}$**	-4.7	-0.9	6.9	10.4
	**$\delta^{18}O_{cell}$ with Peclet**	20.2	7.2	-1.9	-2.0
	$\delta^{18}O_{es}$	0.0	0.0	0.0	0.0
	E	-88.8	-47.3	26.3	40.0
	**g_c**	-89.0	-47.8	25.4	37.7
	**GPP**	-91.5	-52.7	39.5	70.3
	**LAI**	-89.2	-48.4	32.3	55.1
FR	**BAI**	-83.3	-49.5	52.1	98.5
	$\delta^{13}C_{cell}$	6.0	2.7	-1.7	-2.8
	**$\delta^{18}O_{cell}$ with Peclet**	11.1	3.9	-1.5	-2.0
	$\delta^{18}O_{es}$	0.0	0.0	0.0	0.0
	E	-63.2	-29.6	18.6	29.4
	**g_c**	-63.7	-30.0	18.2	28.2
	**GPP**	-57.3	-24.4	12.5	18.2
	**LAI**	-64.2	-30.6	21.7	37.0
g_{cmax}	**BAI**	25.4	12.7	-13.4	-27.6
	**$\delta^{13}C_{cell}$**	21.7	8.0	-5.1	-8.6
	$\delta^{18}O_{cell}$ with Peclet	5.6	2.3	-1.2	-1.7
	$\delta^{18}O_{es}$	0.0	0.0	0.0	0.0
	E	-40.1	-18.9	13.8	23.1
	**g_c**	-40.5	-19.2	13.5	22.4
	GPP	-1.0	0.7	-4.1	-9.8
	**LAI**	-1.9	0.3	-3.6	-8.8
k_g	**BAI**	84.7	40.6	-53.2	-81.7
	$\delta^{13}C_{cell}$	-2.8	-1.7	3.5	6.7
	**$\delta^{18}O_{cell}$ with Peclet**	-0.5	-1.3	6.3	14.7
	$\delta^{18}O_{es}$	0.0	0.0	0.0	0.0
	E	44.3	28.8	-44.4	-75.0
	**g_c**	36.4	24.7	-43.8	-74.3
	**GPP**	29.2	19.9	-38.4	-69.9
	**LAI**	35.8	22.0	-38.3	-69.0
Maximum ASW	**BAI**	-31.2	-11.7	9.3	14.8
	$\delta^{13}C_{cell}$	0.9	0.3	-0.1	0.0
	$\delta^{18}O_{cell}$ with Peclet	1.9	0.6	-0.2	-0.1
	$\delta^{18}O_{es}$	0.0	0.0	0.0	0.0
	E	-12.3	-3.9	1.0	0.4
	**g_c**	-13.5	-4.5	1.3	0.7

	**GPP**	-10.7	-3.5	0.9	0.4
	LAI	-9.2	-2.9	0.6	-0.1
pfs20	**BAI**	-92.5	-50.5	-3.5	-19.6
	$\delta^{13}C_{cell}$	9.9	5.8	-2.9	-4.6
	**$\delta^{18}O_{cell}$ with Peclet**	22.7	10.2	-2.0	-2.0
	$\delta^{18}O_{es}$	0.0	0.0	0.0	0.0
	E	-93.9	-59.8	29.7	42.9
	**g_c**	-94.0	-60.3	28.5	40.3
	**GPP**	-92.2	-53.6	17.8	22.5
	**LAI**	-94.2	-60.8	37.4	60.8
p_{rx}	**BAI**	458.8	91.5	-100.0	-100.0
	**$\delta^{13}C_{cell}$**	-10.6	-7.0	10.7	32.4
	**$\delta^{18}O_{cell}$ with Peclet**	-1.2	-1.7	26.3	26.3
	$\delta^{18}O_{es}$	0.0	0.0	0.0	0.0
	E	71.6	56.3	-100.0	-100.0
	**g_c**	78.8	54.3	-99.9	-99.9
	**GPP**	24.5	24.7	-100.0	-100.0
	**LAI**	169.5	96.8	-100.0	-100.0
wSx1000	**BAI**	86.9	34.5	-18.3	-32.6
	$\delta^{13}C_{cell}$	1.1	0.5	-0.3	-0.6
	$\delta^{18}O_{cell}$ with Peclet	1.4	0.5	-0.4	-0.6
	$\delta^{18}O_{es}$	0.0	0.0	0.0	0.0
	E	-12.6	-5.1	3.6	6.5
	**g_c**	-12.7	-5.2	3.6	6.4
	GPP	-9.8	-3.9	2.6	4.6
	**LAI**	-13.3	-5.5	4.0	7.1

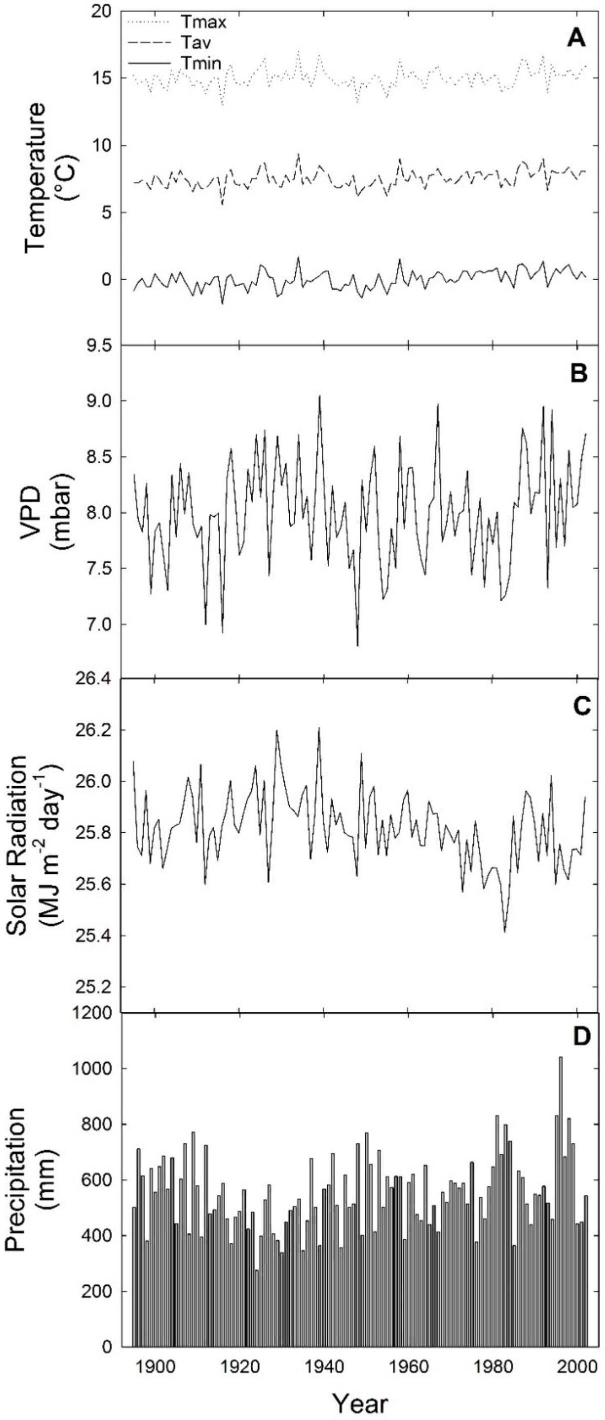

Figure S1. Climate inputs from 1895-2002 at the Metolius site: average annual minimum temperature (T_{min}), maximum temperature (T_{max}), average temperature (T_{av}), vapor pressure deficit (VPD), solar radiation, and precipitation. These average annual values are based on average monthly values that are used in 3-PG.

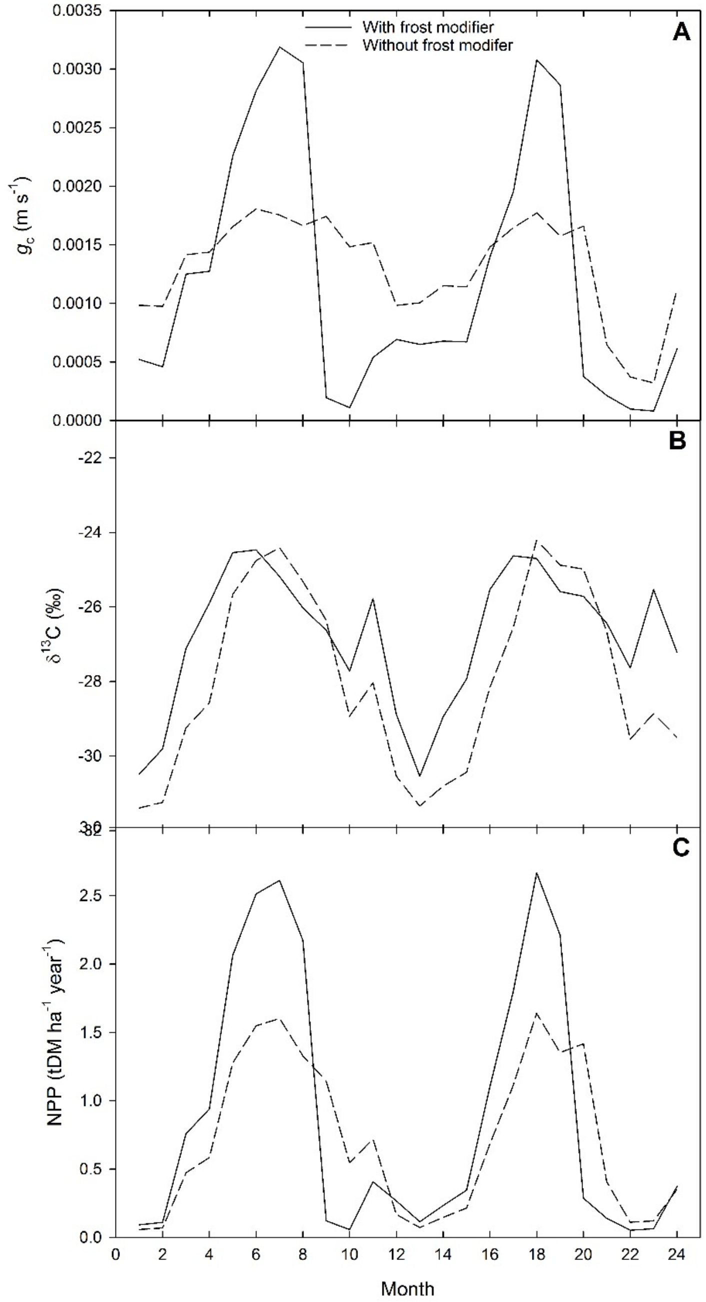

Figure S2. The effect of the addition of the frost modifier for calculating canopy conductance (g_c) to account for zero conductance on days with frost (where minimum temperature <0°C) on g_c, $\delta^{13}C_{cell}$, and net primary productivity (NPP) in 2001-2002. The addition of the frost modifier in the calculation of g_c caused g_c to become lower compared to that without the frost modifier in fall, winter, and spring months with values reaching 0 in winter. This also resulted in greater $\delta^{13}C_{cell}$ in fall, winter, and spring months compared to $\delta^{13}C_{cell}$ without the frost modifier. The inhibition of g_c during winter months also coincided with minimal NPP.

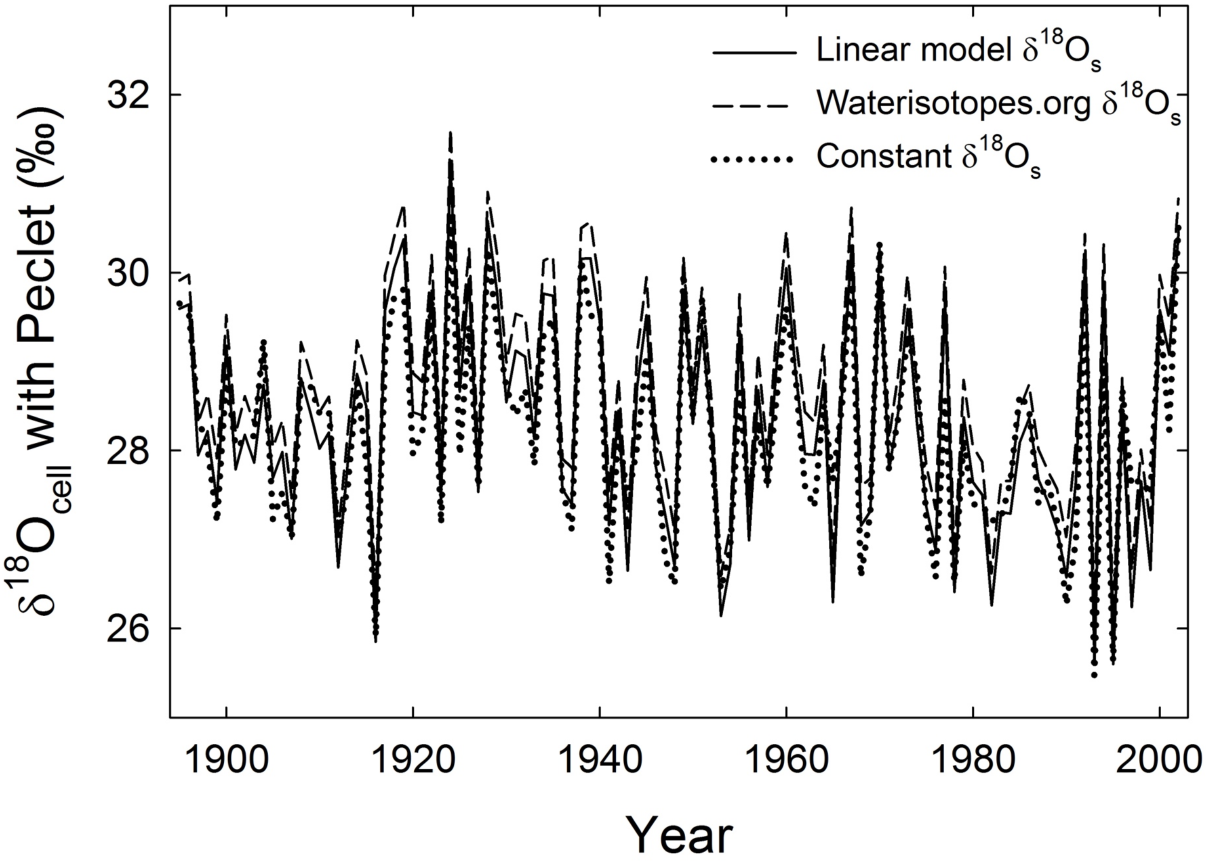

Figure S3. Using variable versus constant $\delta^{18}O$ of soil water ($\delta^{18}O_s$) has little impact on $\delta^{18}O_{cell}$ with Peclet. Variable $\delta^{18}O_s$ was determined from a linear model using monthly temperature and precipitation values obtained from PRISM (see Methods) so $\delta^{18}O_s$ values varied each year from 1895-2002. Constant $\delta^{18}O_s$ was determined by averaging monthly $\delta^{18}O_s$ for 1895-2002 so the same 12 monthly $\delta^{18}O_s$ values were used each year from 1895-2002.

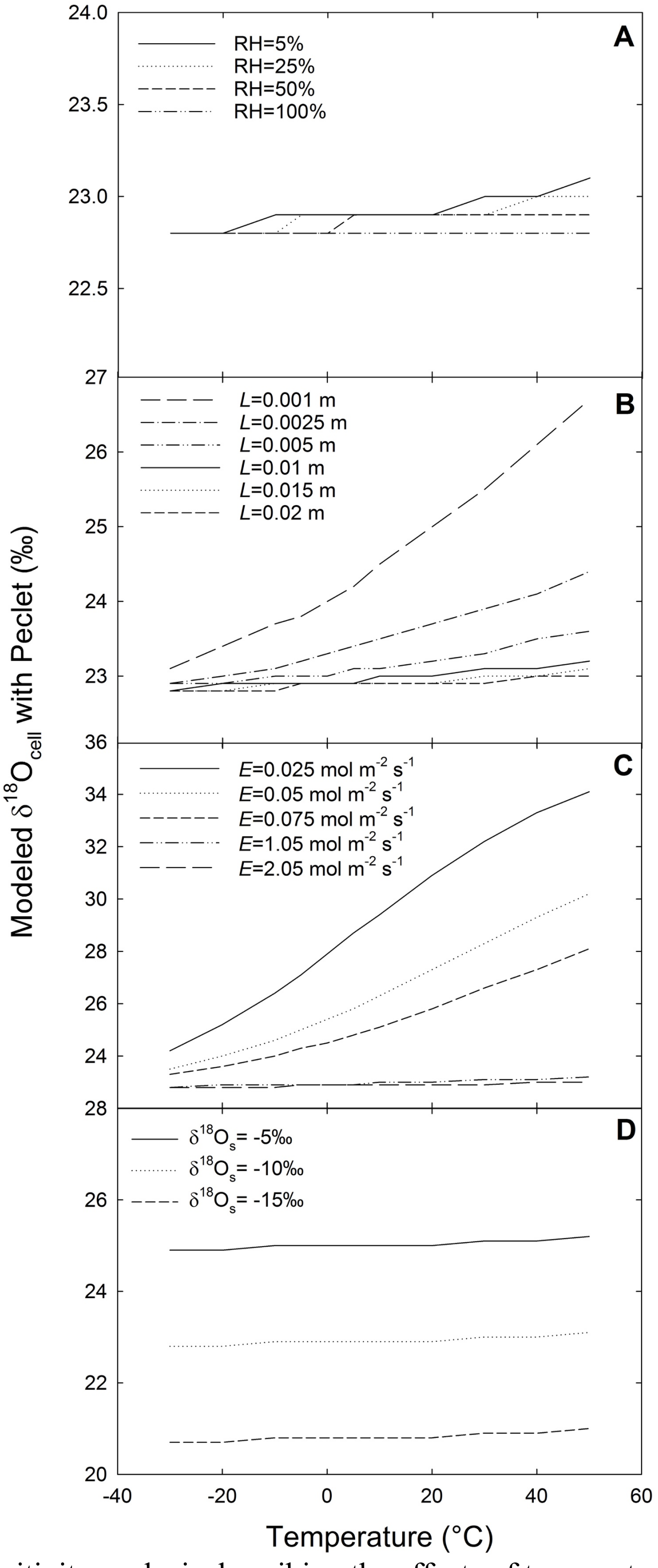

Figure S4. Simple sensitivity analysis describing the effects of temperature, relative humidity (RH), effective path length (L), transpiration (E), and $\delta^{18}O$ of soil water ($\delta^{18}O_s$) on $\delta^{18}O_{cell}$ with Peclet.
