# Supplementary material for: Investigating old‐growth ponderosa pine physiology using tree‐rings, δ13C, δ18O, and a process‐based model
Source: Ecology. 2019 Apr 15;100(6):e02656. doi: 10.1002/ecy.2656 (PMC6645703; doi:10.1002/ecy.2656)
Supplement: Supplementary file 3 [file ECY-100-na-s003.pdf]

Supporting Information. Ulrich, Danielle E. M., Christopher Still, J. Renée Brooks, Youngil Kim, Frederick C. Meinzer. 2019. Investigating old-growth ponderosa pine physiology using tree-rings, $\delta^{13}C$, $\delta^{18}O$, and a process-based model. *Ecology*.

Data S1

Descriptive title of the data and/or code.
3-PG model with d13C and d18O submodels in Python

Authors

Name: Christopher Still
Affiliation: Oregon State University
Address: Christopher Still
Forest Ecosystems and Society
Oregon State University
321 Richardson Hall
Corvallis, OR 97331-5752 USA
Email: chris.still@oregonstate.edu

Name: Hang Zhou
Affiliation: Descartes Lab
Address: Hang Zhou
Descartes Labs, Inc.
1613 Paseo De Peralta Ste. 200
Santa Fe, NM 8750, USA
Email: Joeyzhou1984@gmail.com

Name: Liang Wei
Affiliation: Lanzhou University
Address: Liang Wei
College of Earth and Environmental Sciences
Lanzhou University
222 S Tianshui Rd, Lanzhou, Gansu, 730000 China
Email: liangwei@alumni.uidaho.edu

Name: John Marshall
Affiliation: Swedish University of Agricultural Sciences
Address: John Marshall
Department of Forest Ecology and Management
Swedish University of Agricultural Sciences
Skogmarksgränd, Umeå 90736, Sweden

Email: john.marshall@slu.edu

Name: Danielle E. M. Ulrich
Affiliation: Los Alamos National Laboratory
Address: Danielle E. M. Ulrich
P.O. Box 1663 MS M888
Los Alamos National Laboratory
Los Alamos, NM 87545 USA
Email: daniellem@lanl.gov

File list (files found within DataS1.zip)

```
test/Test_config.cfg
test/Test_output.txt
test/Test_input.txt
lib/__init__.py
lib/BiomassPartition.py
lib/BiomassPartition.pyc py
lib/CanopyProduction.py
lib/CanopyProduction.pyc
lib/constants.py
lib/constants.pyc
lib/framework.py
lib/framework.pyc
lib/Model3PG.py
lib/Model3PG.pyc
lib/StemMortality.py
lib/StemMortality.pyc
lib/Test_config_SCI.cfg
lib/tmp.py
lib/utils.py
lib/utils.pyc
lib/WaterBalance.py
lib/WaterBalance.pyc
bin/3PG.py
data/default.cfg
```

Description

This is the Python version of the 3-PG model with the d13C and d18O submodel. The model itself is primarily found in the '`lib`' folder. The '`test`' folder contains files where specific site, climate, and species information can be adjusted. Variable descriptions are annotated in all files and can be read in a text editor.

`test/Test_config.cfg` - main configuration file where variables describing site and species are adjusted. This file is an example configuration file.
`test/Test_output.txt` - where model outputs are saved to

`test/Test_input.txt` - the file that contains site-specific climate information (e.g. from PRISM). This file is an example input file.
`lib/__init__.py` - needed in this folder to consider this a Python package
`lib/BiomassPartition.py` - biomass partition module
lib/`BiomassPartition.pyc py` - biomass partition module for Python
`lib/CanopyProduction.py` - canopy production module
`lib/CanopyProduction.pyc` - canopy production module for Python
`lib/constants.py` - constants module
`lib/constants.pyc` - constants module for Python
`lib/framework.py` - framework
`lib/framework.pyc` - framework for Python
`lib/Model3PG.py` - 3PG model
`lib/Model3PG.pyc` - 3PG model for Python
`lib/StemMortality.py` - stem mortality module
`lib/StemMortality.pyc` - stem mortality module for Python
`lib/Test_config_SCI.cfg` - sample configuration file
`lib/tmp.py` - subroutine calculations
`lib/utils.py` - utilities
`lib/utils.pyc` - utilities for Python
`lib/WaterBalance.py` - water balance module
`lib/WaterBalance.pyc` - water balance module for Python
`bin/3PG.py` - 3PG model
`data/default.cfg` - Default model configuration file
